# Biochemical mapping reveals a conserved heme transport mechanism via CcmCD in System I bacterial cytochrome *c* biogenesis

**DOI:** 10.1128/mbio.03515-24

**Published:** 2025-04-01

**Authors:** Alicia N. Kreiman, Sarah E. Garner, Susan C. Carroll, Molly C. Sutherland

**Affiliations:** 1 Department of Biological Sciences, University of Delaware 505902, Newark, Delaware, USA; University of Michigan-Ann Arbor, Ann Arbor, Michigan, USA; University of Pennsylvania, Philadelphia, Pennsylvania, USA

**Keywords:** heme, heme transporter, heme trafficking, cytochrome *c*, cytochrome* c *biogenesis

## Abstract

Heme is a redox-active cofactor for essential processes across all domains of life. Heme’s redox capabilities are responsible for its biological significance but also make it highly cytotoxic, requiring tight intracellular regulation. Thus, the mechanisms of heme trafficking are still not well understood. To address this, the bacterial cytochrome *c* biogenesis pathways are being developed into model systems to elucidate mechanisms of heme trafficking. These pathways function to attach heme to apocytochrome *c*, which requires the transport of heme from inside to outside of the cell. Here, we focus on the System I pathway (CcmABCDEFGH) which is proposed to function in two steps: CcmABCD transports heme across the membrane and attaches it to CcmE. HoloCcmE then transports heme to the holocytochrome *c* synthase, CcmFH, for attachment to apocytochrome *c*. To interrogate heme transport across the membrane, we focus on CcmCD, which can form holoCcmE independent of CcmAB, leading to the hypothesis that CcmCD is a heme transporter. A structure-function analysis via cysteine/heme crosslinking identified a heme acceptance domain and heme transport channel in CcmCD. Bioinformatic analysis and structural predictions across prokaryotic organisms determined that the heme acceptance domains are structurally variable, potentially to interact with diverse heme delivery proteins. In contrast, the CcmC transmembrane heme channel is structurally conserved, indicating a common mechanism for transmembrane heme transport. We provide direct biochemical evidence mapping the CcmCD heme channel and providing insights into general mechanisms of heme trafficking by other putative heme transporters.

**IMPORTANCE:**

Heme is a biologically important cofactor for proteins involved with essential cellular functions, such as oxygen transport and energy production. Heme can also be toxic to cells and thus requires tight regulation and specific trafficking pathways. As a result, much effort has been devoted to understanding how this important, yet cytotoxic, molecule is transported. While several heme transporters/importers/exporters have been identified, the biochemical mechanisms of transport are not well understood, representing a major knowledge gap. Here, the bacterial cytochrome *c* biogenesis pathway, System I (CcmABCDEFGH), is used to elucidate the transmembrane transport of heme via CcmCD. We utilize a cysteine/heme crosslinking approach, which can trap endogenous heme in specific domains, to biochemically map the heme transport channel in CcmCD, demonstrating that CcmCD is a heme transporter. These results suggest a model for heme trafficking by other heme transporters in both prokaryotes and eukaryotes.

## INTRODUCTION

Heme is a redox-active molecule critical for survival across all domains of life, functioning in gas sensing, gas transport, electron transfer, and other cellular processes (e.g., circadian rhythms [1], modulation of transport channels [2, 3], miRNA processing [4, 5], adipogenesis [6]). However, the redox properties that confer heme’s biological importance simultaneously render it cytotoxic (7–10). Consequently, heme homeostasis and trafficking must be tightly regulated. It is generally accepted that dedicated heme transport pathways must exist to prevent toxicity (10–16). For example, in eukaryotes heme transporters such as FLVCRs (17–20), HRGs (21–23) and MRPs (24, 25) have been identified. In bacteria, heme uptake pathways used to scavenge environmental heme such as the Has (26, 27), Phu (26), Shu (28), and Isd (29–31) pathways and heme exporters such as HrtAB (32) and CydDC (33, 34) are known. To date, the designation of heme transporters is based primarily on genetic and phenotypic characterization. However, direct biochemical evidence for heme transport mechanisms is lacking, likely due to tight intracellular regulation of heme and the inherently transient nature of trafficking. Here, we describe the first biochemical mapping of a dedicated heme transport channel in the System I cytochrome *c* biogenesis pathway.

**Fig 1:**
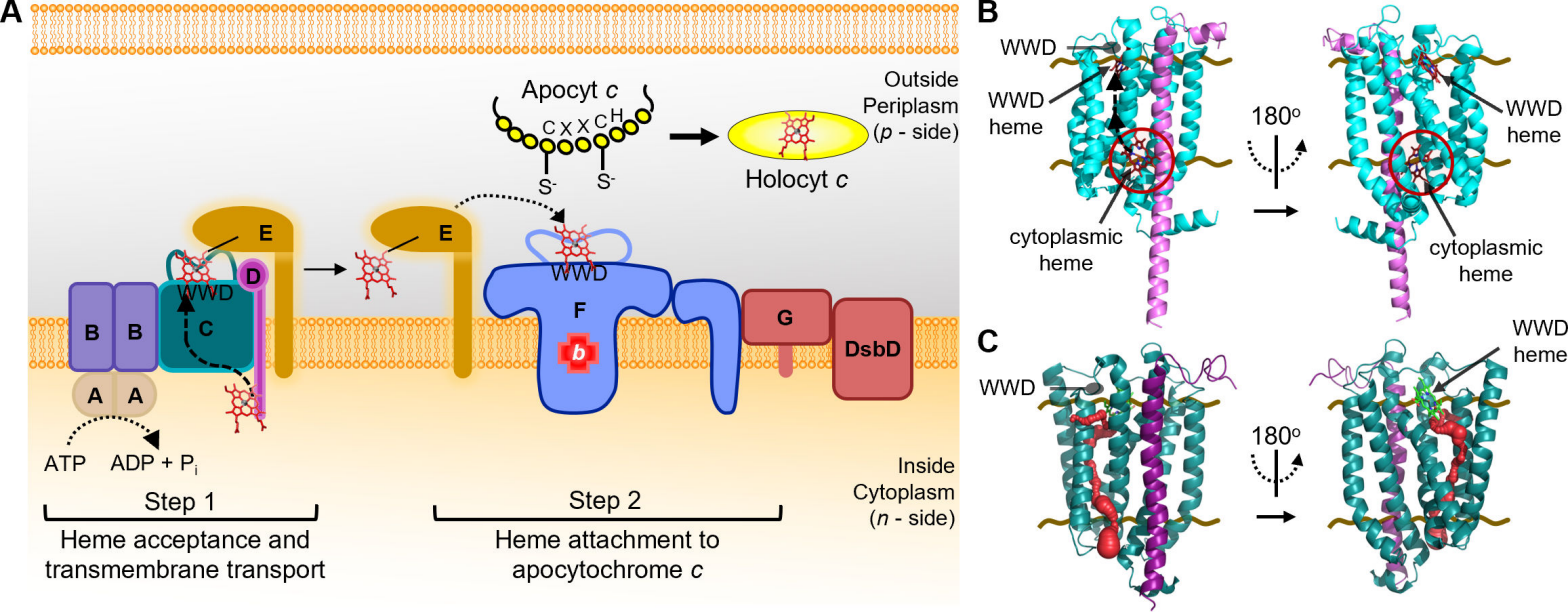
Heme acceptance and transport in the prokaryotic System I cytochrome *c* biogenesis pathway. (A) Model of 2-step *E. coli* System I cytochrome *c* biogenesis pathway (CcmABCDEFGH). Heme acceptance (CcmCD) and transmembrane transport (CcmC) to the CcmC WWD domain is shown. (B) AlphaFold 3 predicted structure of CcmCD (cyan/light purple) + 2 heme molecules (red) displaying the cytoplasmic heme acceptance domain (red circle) and heme transport channel (black arrows) to the WWD domain of CcmC. (C) CAVER 3.0.3 PyMOL plugin model of heme transport channel (red) through CcmCD (PDB 7F04) complex. Standard input values except, probe radius = 0.5 and maximum distance (A) = 5. Input starting point at predicted cytoplasmic heme location. Brown lines indicate inner membrane boundaries.

All cytochromes *c* require the covalent attachment of heme cofactor(s), a process that is mediated by three pathways: System I, composed of CcmABCDEFGH/I and found in α-, γ-proteobacteria, plant and protozoal mitochondria, and archaea. System II, composed of CcsBA and found in Gram (+) bacteria, cyanobacteria, chloroplasts, and ε-proteobacteria. System III, composed of a single enzyme, HCCS, and found in eukaryotic mitochondria (35–40). In all cases, heme attachment occurs in a cellular location distinct from heme biosynthesis; consequently, heme transport across a membrane is required (35, 38, 41). The prokaryotic cytochrome *c* biogenesis pathways are excellent models to interrogate heme chaperoning, modification, and redox, as well as for mapping of discrete heme interaction domains (42–52). Here, we focus on the *Escherichia coli* System I cytochrome *c* biogenesis pathway, composed of eight integral membrane proteins, CcmABCDEFGH, that are proposed to function in two steps (Fig. 1A). In step 1, CcmABCD transport heme across the membrane to the periplasmic CcmC WWD domain (43, 46, 49, 53, 54) where heme is stereospecifically positioned and then covalently attached to CcmE (42, 43, 53–55). ATP hydrolysis via CcmAB mediates the release of holoCcmE (49, 53), which transfers heme to the holocytochrome *c* synthase, CcmFH (55), for attachment to apocytochrome *c* in step 2 (56, 57). Recent studies have identified the stereospecific positioning of heme in the conserved tryptophan-rich periplasmic WWD domains of CcmC (46, 49, 50) and CcmF (51). However, the mechanism of heme delivery to the CcmC WWD domain remains elusive.

CcmC operates in the context of the CcmABCD/E subcomplex during step 1 of cytochrome *c* biogenesis. This subcomplex was first identified over 30 years ago (58, 59) and has been the focus of many genetic, biochemical, and more recently structural studies (42, 43, 45, 46, 49, 50, 53, 54, 60–64). Two recent cryo-EM structures suggest differing stoichiometries for this complex [Ccm(AB)_2_CD (49) vs Ccm(ABCD)_2_/E (50)], but both provide mechanistic insights via multiple cryo-EM conformations, including structural identification of the CcmA ATP binding site and the CcmC WWD heme binding site (49). Yet none of these extensive studies revealed the mechanisms of heme transport. Li et al. suggest the CcmC WWD may receive heme via a cytoplasmic channel or from the upper leaflet of the inner membrane (49). Ilcu et al. propose a heme floppase mechanism at the interface CcmBC (50). Utilizing cysteine/heme crosslinking to trap endogenous heme at multiple positions, we provide biochemical evidence of the CcmCD heme transport channel, thereby determining the mechanism of heme acceptance and transport across the bacterial membrane for System I cytochrome *c* biogenesis.

## RESULTS

### Insights into System I heme transport

To probe the mechanism of heme acceptance and transport across the inner membrane, we focused on step 1 (CcmABCDE) of System I (Fig. 1A). Previous studies demonstrated that in the absence of CcmAB, CcmCDE purified with holoCcmE demonstrating that heme was transported across the membrane and stereospecifically positioned in the CcmC WWD domain to properly form the covalent attachment to CcmE (42, 43, 46, 53, 54). Thus, CcmAB are not required for heme acceptance, heme transport across the membrane to the CcmC WWD domain, nor heme attachment to CcmE at H130 (42, 43, 46, 53, 54, 65–69) (Fig. S1A, B, and D). Subsequent studies in the CcmCDE(H130A) background determined that heme is transported to the CcmC periplasmic WWD domain but not attached to CcmE, providing additional evidence that CcmAB are not involved with heme delivery to the CcmC WWD domain (46, 55, 65) (Fig. S1C and D). Thus, we hypothesized that CcmCD is the heme transporter for System I. Here, we exploit the CcmCDE(H130A) complex to bias heme localization to CcmCD to elucidate the heme acceptance and transport mechanism in System I.

AlphaFold 3 (70) (AF3) was used to predict putative heme interaction domains in CcmCD. In agreement with biochemical (46) and cryo-EM structures (49), AF3 calculations with one heme molecule predicted heme localization in the periplasmic CcmC WWD domain with high confidence pLDDT values (Fig. S2A). Superimposition of the AF3 and CcmCD cryo-EM structure (PDB 7F04) (49) (Fig. S2C, root mean square deviation [RMSD] = 0.754) demonstrates that AF3 can predict heme cofactor localization. AF3 calculations with two heme molecules predicted a second heme interaction domain on the cytoplasmic face of CcmCD (Fig. 1B) with high confidence pLDDT values (Fig. S2B) and similar structural architecture to the CcmCD cryo-EM structure (Fig. S2D, RMSD = 0.777). CAVER 3.0.3 (71) predicted a putative transport channel through CcmC that aligns with the heme locations from AF3 (Fig. 1C).

### Identification of CcmC-heme interactions

We hypothesized that CcmCD functions as a heme transporter. While AF3 and CAVER predicted CcmCD-heme interactions outside the known WWD domain-heme interactions (46, 49, 50) (Fig. 1B and C), direct experimental evidence was needed. A cysteine/heme crosslinking approach was used to test our hypothesis. This approach exploits the natural propensity of cysteine and heme to form a covalent bond (i.e., crosslink) when in proximity (Fig. S3) and has been used to map residues with direct heme interactions in the conserved WWD domain of cytochrome *c* biogenesis proteins CcmC, CcmF, and CcsBA (System II) (46, 47, 51, 52). Cryo-EM structures of CcmABCD and CcsBA have validated these interactions (48, 49), demonstrating the utility of cysteine/heme crosslinking to define heme-protein interactions biochemically.

**Fig 2:**
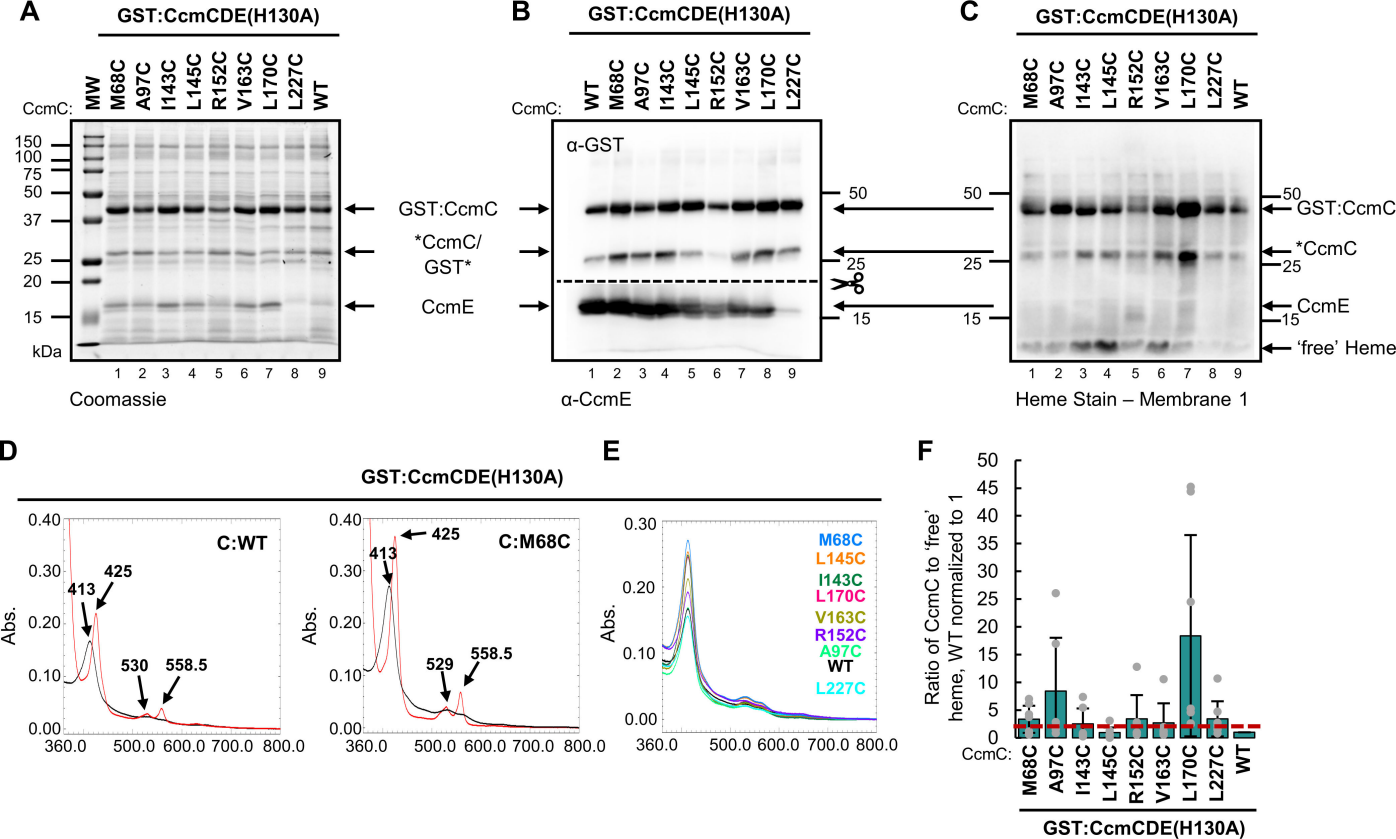
CcmC cysteine/heme crosslink formation demonstrates direct CcmC-heme interactions. Thirty-eight single amino acid cysteine variants were engineered in CcmC. Eight variants formed a cysteine/heme crosslink. (A and B) Stability of the protein complex was analyzed via 15% SDS-PAGE separation followed by (A) Coomassie total protein stain, 5 µg affinity purified protein (B) α-GST and α-CcmE immunoblots, 5 µg affinity purified protein. (C) Heme stain, 10 µg affinity purified protein; representative of three independent affinity purifications. *CcmC indicates cleavage of the n-terminal GST tag from CcmC with crosslinked heme. (D) UV-vis spectral analysis of as purified (black) and sodium dithionite reduced (red) spectra, 50 µg affinity purified protein. Wild-type (WT) and CcmC(M68C) cysteine variant shown as representatives. Data representative of three independent affinity purifications. (E) Heme co-purification determined by relative Soret peak height in as-purified spectral analysis, 50 µg affinity purified protein. CcmC cysteine variant indicated. (F) Quantitation of heme-stained bands: ratio of CcmC bound heme to “free” heme, wild-type ratio normalized to 1. Heme stains were quantified with AzureSpot Software (Azure, v.2.2.167). Black bars represent average ratio of six purifications (gray dots). Error bars indicate standard deviation. A ratio above 2 (red dotted line) indicates the formation of cysteine/heme crosslink.

Thirty-eight single amino acid cysteine variants spanning the cytoplasmic and transmembrane domains of CcmC were engineered in GST:CcmCDE(H130A) (Fig. S4A). Note, no exogenous heme nor manipulation of the *E. coli* heme biosynthesis pathways were used in these studies. Thus, all co-purified heme is endogenously biosynthesized in *E. coli*. CcmC cysteine variants were recombinantly expressed, affinity purified via the n-terminal GST tag and separated by SDS-PAGE to assess protein stability and co-purification with CcmE. All CcmC cysteine variants were stable except G100C (Fig. S4B through D). Cysteine/heme crosslink formation was determined by retention of heme at the CcmC polypeptide (46) via heme stain (72) (Fig. S4E), first by visual assessment, then by quantitative analysis of the CcmC bound to *b*-type “free” heme ratio. Note, most *b-*type or non-covalently bound heme separates from protein polypeptides upon SDS-PAGE and migrates with the dye front as “free” heme. “Free” heme can transfer through the nitrocellulose membrane, so two membranes are layered to capture the total free heme for quantitation (46, 52). GST:CcmCDE(H130A) retains a small amount of *b*-type heme at the CcmC polypeptide (46) (Fig. 2C, lane 9). Thus, the CcmC bound to “free” heme ratio of wild type is normalized to one. A ratio greater than or equal to two indicates the formation of a cysteine/heme crosslink (46). Note, proteolysis of the GST tag occurs in a small population of the GST:CcmC (~53 kDa) affinity purified protein, resulting in GST (26.17 kDa) and CcmC with crosslinked heme (26.81 kDa) polypeptides (Fig. 2B GST, 2C *CcmC).

Initially, 15 of the 38 cysteine variants were selected for further analysis (M68C, I70C, Y71C, A97C, A101C, V102C, F103C, I143C, L145C, H147C, R152C, V163C, N169C, L170C, and L227C) (Fig. S4F, asterisks). Subsequent analysis determined that eight CcmC cysteine variants formed a cysteine/heme crosslink based on a CcmC bound to *b*-type “free” heme ratio greater than 2 (M68C, A97C, I143C, L145C, R152C, V163C, L170C, and L227C) (Fig. 2A through C and F). Cysteine/heme crosslink formation was further validated by quantification of the CcmC heme-stained polypeptide, demonstrating an increase in heme retention at CcmC for cysteine variants compared to wild type (Fig. S5C). CcmC heme retention was not statistically significant for all CcmC cysteine variants in this analysis. However, these variants were still categorized as forming a cysteine/heme crosslink based on the CcmC bound to “free” heme ratio greater than 2. In previous studies of the CcmC and CcsBA WWD domains, a bound to “free” heme ratio of 2 was used to define cysteine/heme crosslink formation. Residues identified by this metric have subsequently been shown to interact with heme in cryo-EM studies (46–49). Thus, the bound to “free” heme ratio is valid for identification of cysteine/heme crosslink formation. Cysteine/heme crosslink formation displayed some variability across biological replicates (Fig. 2F; Fig. S5C); however, given that heme transport is a dynamic, transient process, this was expected.

The heme environment of the CcmC cysteine variants was assessed via UV-vis spectral analysis. The heme environment was unperturbed, and all samples exhibited as-purified Soret peaks similar to wild type at ~413 nm (Fig. 2D, black line, S5A) and sample reduction resulted in a Soret peak shift to ~425 nm with the formation of characteristic β- (~530 nm) and α-peaks (~559 nm) (Fig. 2D, red line, Fig. S5A). Analysis of as-purified Soret peaks revealed that the CcmC cysteine variants co-purify with endogenous heme at levels greater than or equal to wild type (Fig. 2E). To further probe the heme environment, a single pyridine hemochrome was performed. The α-peak of reduced pyridine hemochrome spectra reflects the number of covalent bonds to heme (73). Wild-type CcmC had a reduced pyridine α*-*peak of 556 nm, indicating all co-purified heme is *b*-type and is retained in the CcmC WWD domain as shown previously (46). The CcmC cysteine/heme crosslinking variants have reduced pyridine α-peaks from 556 to 555 nm, indicating that the majority of co-purified heme is *b*-type (Fig. S5B), which we predict is localized to the CcmC WWD domain with a minor fraction of the total heme trapped at the cysteine/heme crosslink. This is similar to results from cysteine/heme crosslinking analyses of CcmF and the System II cytochrome *c* biogenesis protein CcsBA in which pyridine hemochrome assays did not detect formation of the cysteine/heme crosslink due to most co-purified heme being *b*-type (47, 51). UV-vis analysis reflects the average heme environment in the sample; therefore, the inability to detect the minor population of crosslinked heme is not unexpected. These results are consistent with the heme stains, which indicate both “free” and CcmC crosslinked heme (Fig. 2C). Given the transient nature of heme trafficking, it is not surprising that cysteine-crosslinked heme represents a minor component of the total heme population, much of which likely resides in the CcmC WWD domain.

### CcmD interacts with heme

CcmD was proposed to play a role in the release of holoCcmE from CcmABCD as deletion of CcmD impacted the amount of holoCcmE (42, 74). We hypothesized that reduced levels of holoCcmE in the absence of CcmD could indicate a role for CcmD in heme transport. To test this, 13 single amino acid cysteine variants spanning the cytoplasmic, transmembrane, and periplasmic domains of CcmD were engineered in GST:CcmCDE(H130A) (Fig. S4A). Similar to CcmC, recombinant expression and affinity purification of CcmD cysteine variants indicated the proteins are stable and co-purify with CcmE, except G16C (Fig. S4B through D). Formation of CcmD cysteine/heme crosslinks was assessed as described above for CcmC. Seven CcmD cysteine variants were selected for further study (G15C, Y17C, A18C, F19C, Q42C, A45C, and I46C) (Fig. S4F, asterisks) and two CcmD cysteine variants were identified that formed cysteine/heme crosslinks (Y17C, I46C).

**Fig 3:**
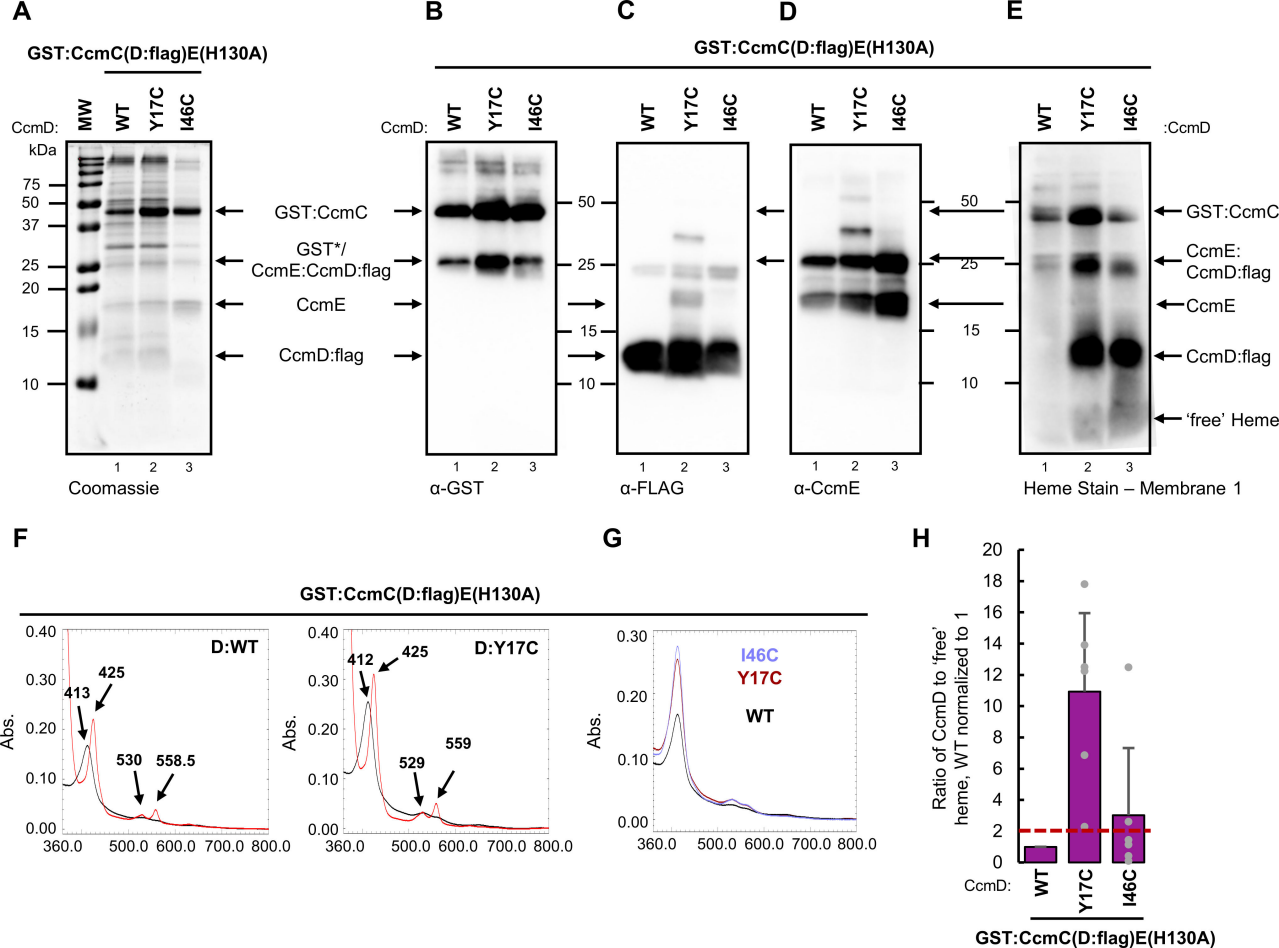
CcmD directly interacts with heme. Thirteen single amino acid cysteine variants were engineered in CcmD. Two variants formed a cysteine/heme crosslink in CcmD. (A–D) A 5 µg of GST affinity purified protein, separated via 12.5% SDS-PAGE and assessed via (A) Coomassie total protein stain, (B) α-GST, (C) α-FLAG, and (D) α-CcmE immunoblots. Representative of three independent affinity purifications. (E) A 10 µg GST affinity purified protein was assessed via heme stain. Representative of three independent affinity purifications. (F) UV-vis spectral analysis of as-purified (black) and sodium dithionite reduced (red) spectra, 50 µg purified protein. Wild-type (WT) and CcmD(Y17C) variant shown. Representative of three independent affinity purifications. (G) Heme co-purification was determined by relative Soret peak height in the purified spectral analysis, 50 µg affinity purified protein. CcmD variant indicated. Representative of three independent purifications. (H) Quantitation of heme-stained bands: ratio of CcmD bound heme to “free” heme, wild-type normalized to 1. Heme stains were quantified with AzureSpot Software (Azure, v.2.2.167). Black bars represent average ratio of six independent purifications (gray dots). Error bars indicate standard deviation. A ratio above 2 (dotted red line) indicates the formation of cysteine/heme crosslink.

To confirm cysteine/heme crosslink formation in CcmD, CcmD:flag fusions were engineered to produce GST:CcmC(D:flag)E(H130A). CcmD cysteine variants in the GST:CcmC(D:flag)E(H130A) background were affinity purified via the n-terminal GST tag and were stable, contained GST and flag tags, co-purified with CcmE and formed cysteine/heme crosslinks (Fig. 3A through E). Cysteine/heme crosslink formation was variable, but CcmD to “free” heme ratios averaged greater than two (Fig. 3E and H). Cysteine/heme crosslink formation was further validated by quantification of the CcmD:flag heme stained polypeptide, demonstrating an increase in heme retention at CcmD (Fig. S6C). Note, GST:CcmC(D:flag)E(H130A) forms a ~25 kDa CcmE-CcmD:flag complex that is not present in GST:CcmCDE(H130A) (compare Fig. 2A and B; 3A, C, and D). To ensure the CcmD:flag did not affect biological function, CcmD:flag was engineered in the complete System I pathway and assayed for levels of cytochrome *c* biogenesis. System I with CcmD:flag produced cytochrome *c* at levels comparable to published System I constructs (75) and thus does not impact biological function (Fig. S7).

The heme environment of the CcmD:flag cysteine variants was assessed via UV-vis spectral analysis and was similar to wild type (Fig. 3F; Fig. S6A). Analysis of the as-purified Soret peak (~413 nm) indicates that CcmD cysteine variants co-purify with increased heme compared to wild type (Fig. 3G). Similar to the analysis of the CcmC cysteine variants (Fig. S5B), a single pyridine hemochrome analysis did not identify robust formation of a covalent bond (Fig. S6B), likely due to the majority of co-purified heme being retained as *b-*type heme in the CcmC WWD domain and a minor population forming the covalent cysteine/heme crosslink in CcmD.

**Fig 4:**
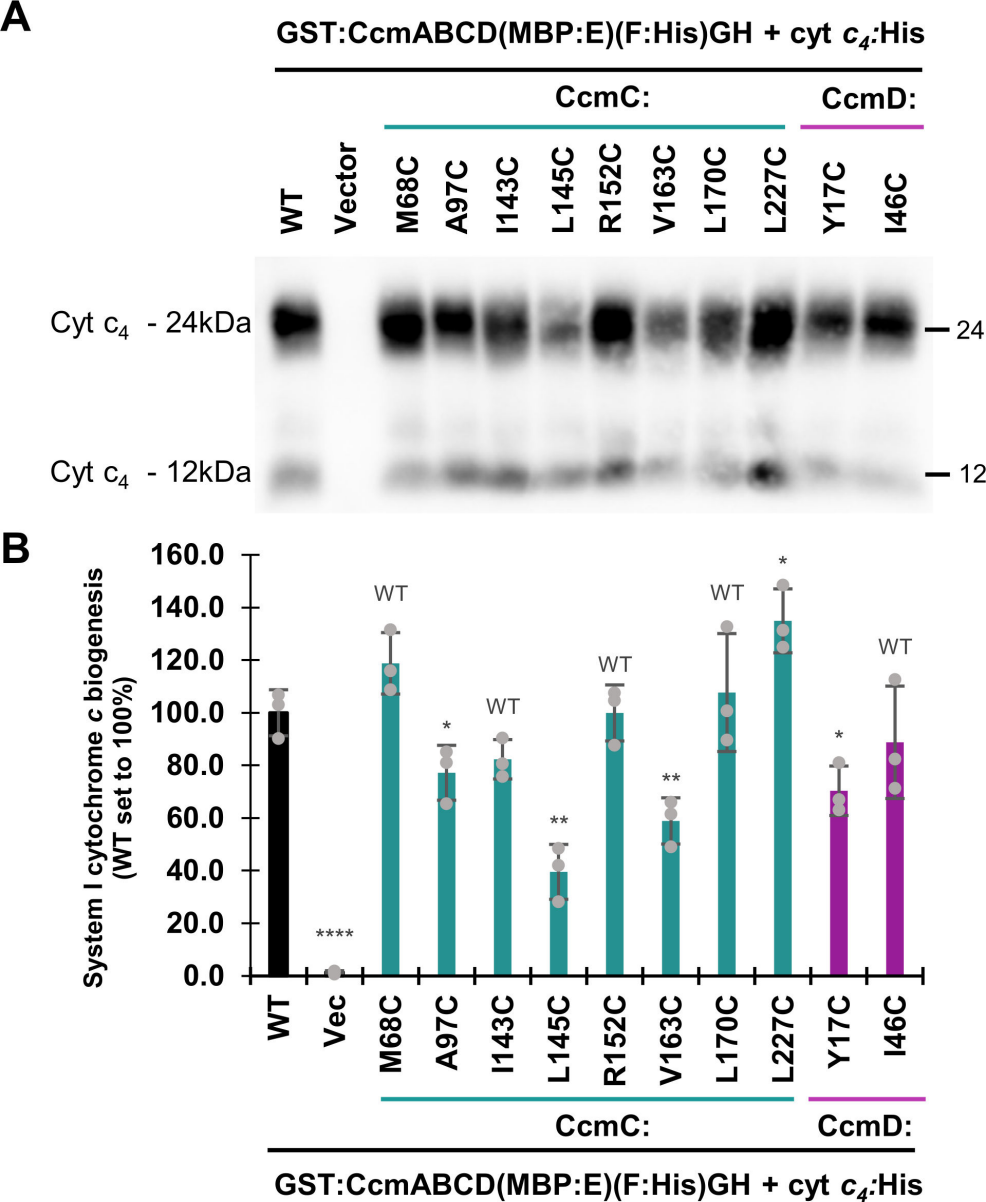
Impact of CcmC/D cysteine/heme crosslinking variants on cytochrome *c* biogenesis. (A) Heme stain of cytochrome *c* biogenesis assay, 50 µg total protein cell lysate. (B) Quantitation of cytochrome *c* biogenesis, bars represent average of three technical replicates, error bars show the standard deviation from the mean, and dots indicate individual data points. Statistical analysis was performed with GraphPad Prism (v10.4.1) using a two-tailed, unpaired *t*-test. Variants that have >80% function are classified as wild type and are thus not statistically significant (WT). *P < 0.05, **P < 0.01, ****P < 0.0001. Heme stains were quantified with AzureSpot Software (Azure, v.2.2.167). Data are representative of three independent biological replicates, each containing three technical replicates.

To determine the impact of the CcmCD cysteine variants on cytochrome *c* biogenesis, the crosslinking residues were engineered in the context of the full System I pathway [GST:CcmABCD(MBP:E)(F:His)GH], which produces holocytochrome *c* at levels similar to the well-characterized GST:CcmABCDE(F:His)GH (43, 51, 56, 75) (Fig. S7). The CcmCD wild-type or cysteine variants were co-expressed with cytochrome *c*_4_:His in *E. coli* Δ*ccm*. The efficiency of cytochrome *c* biogenesis (i.e., heme attachment) of each CcmC or D cysteine variant was assayed via quantitative heme stain (72, 75, 76). Five cysteine variants were classified as wild type for cytochrome *c* biogenesis [>80% of wild-type function; CcmC(M68C, I143C, R152C, L170C); CcmD(I46C)] (Fig. 4A and B; Fig. S8). One variant, CcmC(L227C), had greater than wild-type levels of cytochrome *c* biogenesis. Four variants were partially functional [25%–80% of wild-type function; CcmC(A97C, L145C, V163C); CcmD(Y17C)] (Fig. 4A and B; Fig. S8). All variants co-purified with wild type or higher levels of total heme (Fig. 2E and 3G). Therefore, an inability to accept or interact with heme is not responsible for the observed biogenesis defects. Alternatively, variable amounts of heme may be retained by the cysteine/heme crosslink, resulting in a heterogeneous cellular population with a majority of wild-type complexes and a minority of crosslinked heme-protein complexes. This is supported by the mixed population of crosslinked and *b*-type heme in all cysteine variants (Fig. 2C and 3E) and pyridine hemochrome assays that indicate majority *b*-type heme (Fig. S5B and S6B).

This is particularly relevant as cytochrome *c* biogenesis assays (Fig. 4) are performed in the context of the complete System I pathway in which holoCcmE is formed to deliver heme to CcmFH. Previously, it was proposed that the covalent bond to CcmE H130 is preferred over cysteine/heme crosslink formation (46), thus we anticipate that the population of CcmCD crosslinked heme is further reduced in the cytochrome *c* biogenesis assays. Therefore, we propose that the cytochrome *c* produced in this assay results from the non-crosslinked population.

### The CcmCD heme channel

**Fig 5:**
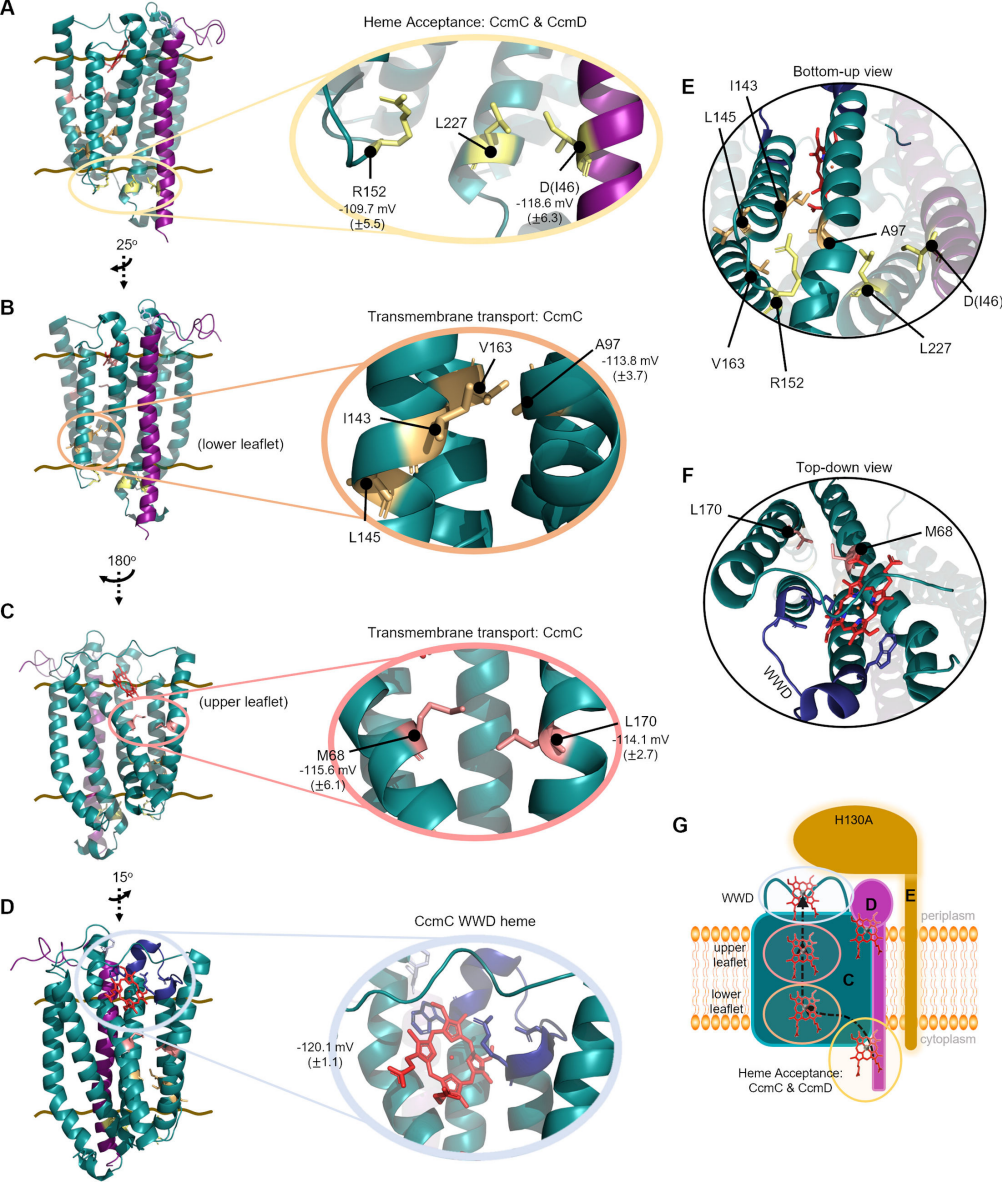
Structural mechanism for heme acceptance and transport across the bacterial inner membrane via CcmCD. All images created from PDB 7F04 in PyMOL (version 2.4.1). Brown lines indicate membrane boundary, cysteine/heme crosslinking variants are labeled, and redox potentials are indicated. (A) Heme acceptance domain at cytoplasmic face with crosslinking residues highlighted inside yellow circle. (B) Heme transport channel domain in lower leaflet of CcmC TMD 2/3/4/5. (C) Heme transport domain in upper leaflet of CcmC TMD 2/3/4/5. (D) Wild-type WWD domain of CcmC. (E) Bottom-up and (F) top-down views of cysteine/heme crosslinking residues spanning the domain of heme movement through CcmCD (PDB 7F04). (G) Schematic of heme transport via CcmCD. The heme interaction domains identified by cysteine/heme crosslinking are indicated and colored as in panels A to D. Heme movement is indicated with dashed arrows. Teal—CcmC, purple—CcmD, gold—CcmE, red—heme.

**Fig 6:**
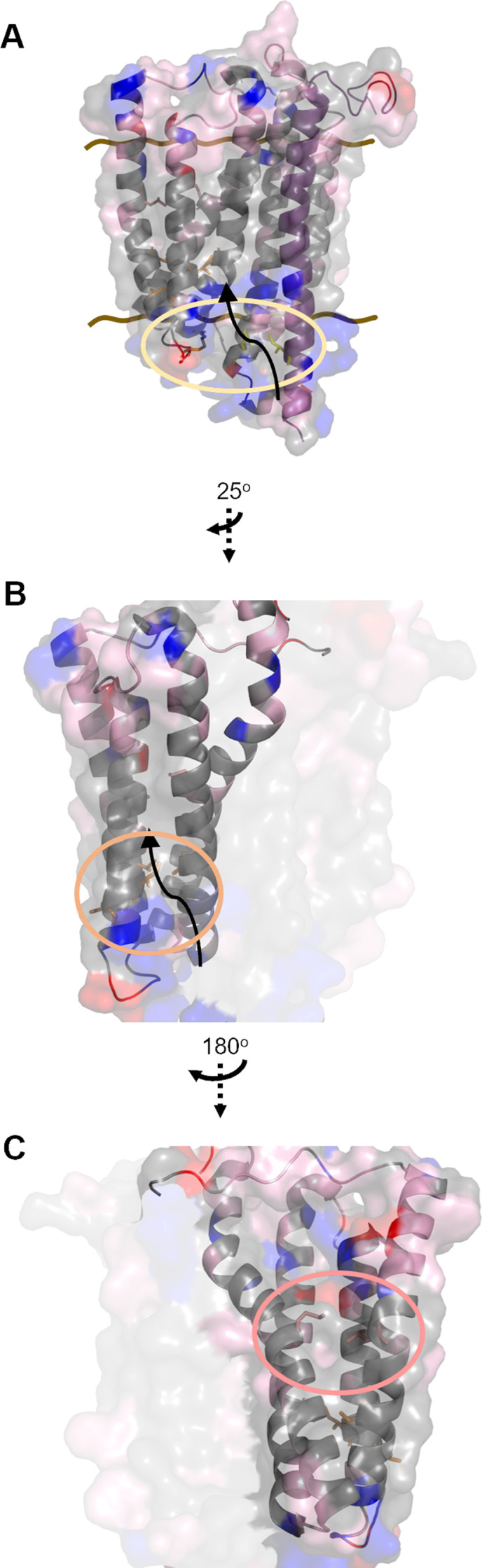
Hydrophobicity of CcmCD heme channel drives heme transport. All images created from PDB 7F04. Brown lines indicate membrane boundary, cysteine/heme crosslinking variants are shown by amino acid structure, arrows indicate the path of heme transport. Hydrophobicity of amino acid residues is indicated as follows: hydrophobic—gray, polar—pink, negatively charged—red, positively charged—blue. Hydrophobicity was determined in PyMOL (version 2.4.1). (A) Heme acceptance domain circled. (B and C) Core transport domain TMD 2/3/4/5. (B) Lower leaflet crosslinking residues circled in orange. (C) Upper leaflet crosslinking residues circled in red. Black arrows indicate the proposed path of heme transport.

The cysteine/heme crosslinking residues were mapped on the CcmCD cryo-EM structure (PDB 7F04) (49) identifying a heme acceptance domain and heme channel (Fig. 5). We propose a model for heme transport from the cytoplasm to the periplasmic CcmC WWD domain based on cysteine/heme crosslink formation. First, heme is delivered to the heme acceptance domain comprised of CcmC transmembrane domains (TMD) 4/5/6 (encoding R152C and L227C) and CcmD(I46C) on the cytoplasmic face of the proteins (Fig. 5A). After acceptance, heme is transferred to the transmembrane heme transport channel composed of CcmC TMD 2/3/4/5. The heme channel is defined by two major clusters of cysteine/heme crosslinking residues. The lower channel cluster is composed of CcmC cysteine variants A97C, I143C, L145C, and V163C (Fig. 5B). The upper channel cluster consists of CcmC cysteine variants M68C and L170C (Fig. 5C). The mapped heme channel is positioned just below the CcmC WWD domain where heme is stereospecifically positioned for attachment to CcmE (46, 49, 50) (Fig. 5D). Further analysis of the heme acceptance domain and heme channel revealed that they are primarily hydrophobic (Fig. 6). The hydrophobic nature of the heme channel is consistent with analyses of heme interaction motifs in other hemoproteins (77, 78). CcmD Y17C is positioned in the periplasm, away from the CcmC WWD domain and transmembrane heme transport channel (Fig. S9) which may indicate a distinct role for the periplasmic CcmD heme interaction domain.

To further probe heme transport through CcmCD, heme redox potentials for the cysteine/heme crosslinking variants with the largest CcmC/D bound to “free” heme ratio in each cluster were determined via a modified Massey method (79–81). Previous analysis of heme redox potentials in protein subcomplexes of System I indicate that heme redox state changes during heme trafficking by System I. Heme is initially reduced when associated with CcmCDE, then oxidized for covalent attachment to CcmE and subsequently re-reduced in CcmFH before attachment to apocytochrome *c* (44, 45, 49, 82). The published redox potential of −120.1 mV for GST:CcmCDE(H130A) (45) was replicated, and CcmD:flag did not impact the redox potential (Fig. S10). The CcmCD cysteine/heme crosslinking variants in the heme acceptance domain and heme channel had slightly reduced redox potentials, ranging from −109 mV to −115 mV (Fig. 5; Fig. S10). Thus, heme remains reduced in the CcmCD heme channel. CcmD Y17C resides outside the heme channel and had a redox potential of −131 mV (Fig. S10). A caveat is that the redox potential represents an average of the total heme in the protein, so it is representative of a heterogeneous protein population of crosslinked heme in the transport domains and *b*-type heme in the CcmC WWD domain. Notably, these results suggest that transmembrane heme transport via CcmCD is similar to System II, CcsBA, where heme is protected from oxidation during transmembrane transport (83, 84).

### Structural conservation of the CcmCD heme channel

**Fig 7:**
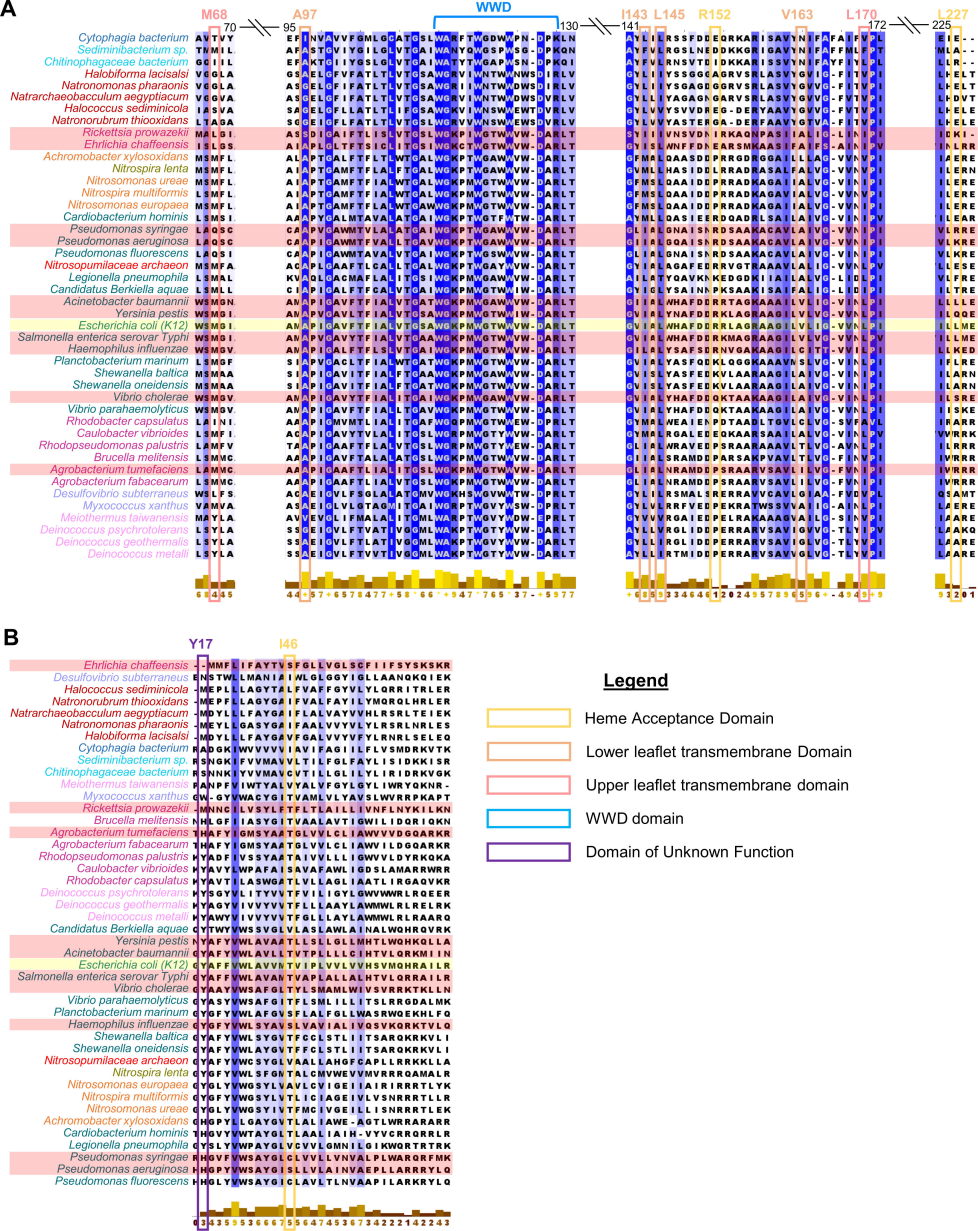
Conservation of CcmC/D crosslinking residues assessed across *E. coli* and 43 organisms containing System I. Multiple sequence alignment (MSA) of CcmC and CcmD amino acid sequences. Sequences aligned utilizing Clustal O (1.2.4) software through the UniProt interface and output determined from alignment. *E. coli* CcmC (highlighted in yellow) represents model organism for this study. Conservation per residue calculated in JalView software (v2.11.4.1) and residues are colored by level of conservation >5 (light blue) to fully conserved (royal blue); * indicates fully conserved residue; + indicates all 10 properties analyzed were conserved. Partial portions of alignment surrounding crosslinking residues are displayed, and crosslinking residues are outlined based on heme transport class defined in Fig. 5 [heme acceptance domain (yellow) C(R152, L227) and D(I46), lower leaflet transmembrane domain (orange) C(A97, I143, L145, V163), upper leaflet transmembrane domain (red) C(M68, L170), periplasmic domain of unknown function (purple) D(Y17)]. The highly conserved WWD domain is indicated with the blue bracket. Taxonomic class is indicated by color coding: γ-proteobacteria (teal), β- (orange), α- (maroon), δ- (light purple), Cytophagia (royal blue), Chitinophagia (light blue), Halobacteria (dark red), Nitrospira (gold), Nitrososphaeria (bright red), Deinococci (light pink). Organisms highlighted in red are included in Table 1. (A) CcmC MSA. (B) CcmD MSA.

To evaluate potential conservation of the heme channel, CcmC and CcmD sequences from 44 organisms representing the taxonomic classes that encode System I (Table S1) were analyzed via Clustal O (85) and JalView (86). *E. coli* CcmC exhibited sequence similarities ranging from 86.94% (*Salmonella enterica* serovar Typhi) to 19.16% (*Sediminibacterium* sp.) (Fig. S11). *E. coli* CcmD exhibited sequence similarities ranging from 75.36% (*Salmonella enterica* serovar Typhi) to 7.55% (*Desulfovibrio subterraneus*) (Fig. S12). While overall percent identity was low, JalView analysis revealed the CcmC WWD domain was highly conserved across all organisms, and three WWD residues identified to form cysteine/heme crosslinks (*E. coli* CcmC W114, D126, and R128) (46) were among the most conserved (Fig. 7A; Fig. S13A). Five (A97, I143, L145, V163, and L170) of seven CcmC heme channel cysteine/heme crosslinking residues had JalView scores above 5, indicating conservation of heme channel residues (Fig. 7A) (86). Both analyses revealed that CcmD was more variable in sequence conservation and protein size (e.g., *Deinococcus psychrotolerans*, 37 amino acids; *Yersinia pestis*, 100 amino acids) (Fig. 7B; Fig. S13B). The CcmD cysteine/heme crosslinking residues were not conserved.

**Fig 8:**
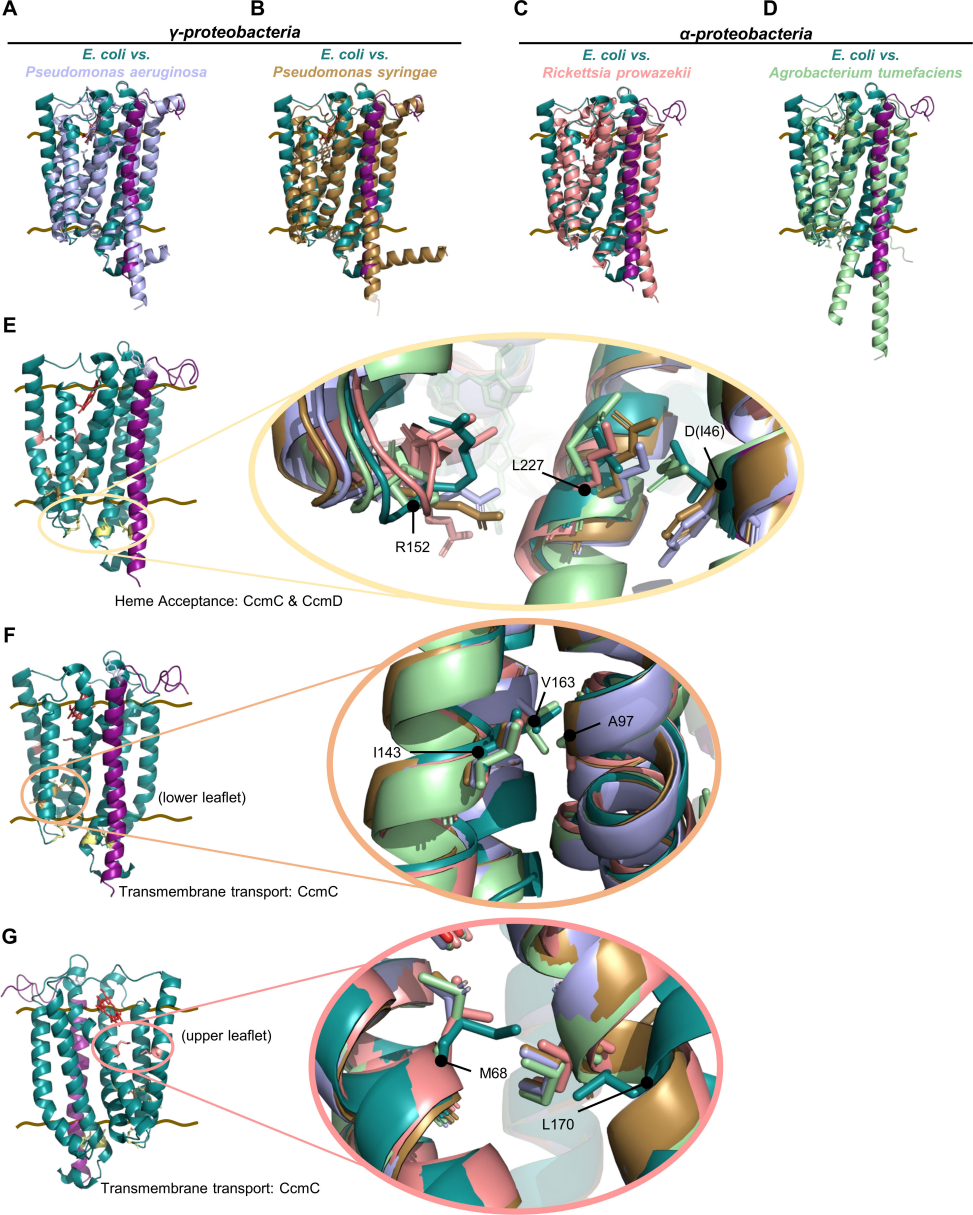
Structural comparisons of CcmCD heme acceptance and transport channel from *E. coli* and four pathogens. (A–D) AlphaFold 3 structural prediction models of CcmCD for (A) *P. aeruginosa,* (B) *P. syringae,* (C) *R. prowazekii,* and (D) *A. tumefaciens* individually superimposed with *E. coli* CcmCD (PDB 7F04) in PyMOL (version 2.4.1). Brown lines indicate membrane boundaries. (E–G) Full view representative structure of *E. coli* CcmCD (PDB 7F04). Zoom in views with all organisms' AF3 structures superimposed with *E. coli* CcmCD (PDB 7F04) (colored in accordance with A–D). (E) Heme acceptance domain at cytoplasmic face with crosslinking residues inside yellow circle and labeled in accordance with *E. coli* amino acid sequence. (F) Heme transport channel domain in lower leaflet of CcmC TMD 2/3/4/5. Crosslinking residues displayed as sticks in orange circle. (G) Heme transport domain in upper leaflet of CcmC TMD 2/3/4/5. Crosslinking residues displayed in salmon circle.

To determine if the CcmCD heme channel was structurally conserved despite the lower overall sequence homology, key proteobacteria that encode System I were analyzed (Table S1, red highlights). Proteobacteria encompass the major portion of organisms that encode System I (35) and are one of the largest, most diverse divisions of prokaryotes. Proteobacteria also include a large number of pathogens (87). AF3 structure determination and superimposition with *E. coli* CcmCD cryo-EM structure (PDB 7F04) (49) was used to determine heme channel structural conservation of representative agriculture pathogens or human pathogens that are listed in the World Health Organization priority pathogen list (88) or the National Institute of Allergy and Infectious Disease biodefense pathogen list (89) (Table 1). Pathogens with low sequence homology to *E. coli* CcmC and CcmD were interrogated, such as γ-proteobacteria human pathogen *Pseudomonas aeruginosa* (Fig. 8A) and plant pathogen *Pseudomonas syringae* (Fig. 8B); α-proteobacteria human pathogen *Rickettsia prowazekii* (Fig. 8C) and plant pathogen *Agrobacterium tumefaciens* (Fig. 8D). AF3 predicted structures were superimposed on *E. coli* CcmCD (PDB 7F04) (49) for analysis of residues homologous to the cysteine/heme crosslinking residues (Fig. 8E through G). The heme acceptance domain has variability in the positioning of the crosslinking residues (Fig. 8E), indicating that heme acceptance by System I could differ among bacteria. In contrast, the transmembrane clusters are highly conserved (Fig. 8F and G). This demonstrates that despite low sequence homology, the heme transport channel is structurally conserved, thus there is a conserved mechanism of heme transport across the bacterial inner membrane by System I.

**TABLE 1:** Structural similarity of proteobacteria CcmCD

Class^*a*^	Organism	Pathogen				CcmC similarity (%)^*d*^	CcmD similarity (%)^*e*^	RMSD^*f*^
		Human	Plant	WHO^*b*^	Biodefense^*c*^			
α	*Agrobacterium tumefaciens*		X^*g*^			45.1	21.8	1.095
α	*Ehrlichia chaffeensis*	X			X	40.3	15.9	2.086
α	*Rickettsia prowazekii*	X			X	40.5	11.6	1.360
γ	*Acinetobacter baumannii*	X		Critical		74.3	55.1	0.867
γ	*Haemophilus influenzae*	X		Medium		59.1	35.8	1.019
γ	*Pseudomonas aeruginosa*	X		High		49.0	29.3	1.307
γ	*Pseudomonas syringae*		X			49.8	31.0	1.147
γ	*Salmonella enterica* serovar Typhi	X		High	X	86.9	75.4	0.786
γ	*Vibrio cholerae*	X			X	63.3	37.3	0.914
γ	*Yersinia pestis*	X			X	75.1	50.7	0.851

^*a*^ Proteobacteria class.

^*b*^ WHO bacterial priority pathogens list, 2024.

^*c*^ NIAID Biodefense Pathogens (89) and CDC Bioterrorism Agents/Diseases.

^*d*^ Amino acid sequence percent identity compared to *E. coli* (see Fig. S11).

^*e*^ Amino acid sequence percent identity compared to *E. coli* (see Fig. S12).

^*f*^ Root mean square deviation calculated in PyMOL for superimposition of AF3 predicted structure and CcmCD (PDB 7F04).

^*g*^ X indicates organism classification as human or plant pathogen and if organism is included in the Biodefense Pathogens list.

## DISCUSSION

Here, we address the long-standing question of heme transport from the cytoplasm across the bacterial membrane to the CcmC WWD domain by System I. It is well established that in the absence of CcmAB, heme is stereospecifically localized in the CcmC WWD domain and holoCcmE is properly formed (42, 46, 53, 54). The data presented here, coupled with the formation of holoCcmE in the absence of CcmAB (42, 46, 53, 54), do not support a role for CcmB in heme acceptance for System I (90) or the proposition that CcmB is responsible for transmembrane heme transport (50, 91). Rather, our biochemical evidence using cysteine/heme crosslinking demonstrates that CcmCD has a cytoplasmic heme acceptance domain and an enclosed heme channel spanning the bacterial membrane leading to the WWD domain (Fig. 2C, 3E, and 5). Thus, CcmCD is likely the heme transporter for System I. The cytoplasmic heme acceptance domain is comprised of CcmC TMD 4/5/6 and CcmD (Fig. 5A and E). After delivery, heme is moved to the CcmC heme channel composed of TMD 2/3/4/5 (Fig. 5B, C, and F) and trafficked to the WWD domain (Fig. 5D) for stereospecific positioning and subsequent attachment to CcmE (46, 49) (Fig. 5G; Movie S1).

The mechanism of heme delivery to CcmCD is unknown. Recent molecular simulations of heme acceptance by the putative heme transporter CydDC and models for CcmABCD suggest heme could be delivered directly from the membrane (34, 50, 91). We do not favor this model for CcmCD due to heme cytotoxicity. Instead, we support a heme chaperone delivery model whereby heme is delivered to a hemoprotein or heme transporter from either the terminal enzyme in heme biosynthesis or from the labile heme pool (11, 16). In both prokaryotes and eukaryotes, there is growing evidence of soluble heme chaperones with roles in heme trafficking (e.g., TANGO2, GAPDH, and HemW) (reviewed in references 16, 91, 92). While the mechanisms of intracellular heme trafficking are still not well understood, the structural diversity observed in the CcmCD heme acceptance domain (Fig. 8E) suggests that System I may require flexibility to accept heme from multiple delivery proteins. Heme delivery via varied heme chaperones would allow for System I to ensure heme availability for cytochrome *c* biogenesis, a process critical to bacterial bioenergetics.

After delivery, heme is moved to the CcmC heme channel composed of TMD 2/3/4/5. These four TMDs comprise the WWD “core region” (46, 49), which is present in other WWD encoding heme-handling proteins, CcmF (51) and CcsBA (47, 48, 93). Heme interacting residues were identified that span the transmembrane region (Fig. 5B and C), with the side chains of cysteine/heme crosslinking residues delineating an enclosed protein channel for heme delivery to the CcmC WWD domain (Fig. 5E through G). Conservation of the heme channel structural architecture across proteobacteria (Fig. 8F and G) indicates that post-delivery, the mechanism of heme transport to the WWD domain is conserved.

None of the CcmD cysteine variants located in the transmembrane domain crosslinked heme. Therefore, CcmD is specifically involved with heme acceptance but not transmembrane transport. Interestingly, the second CcmD crosslinking variant, Y17C, is located in the periplasmic space (Fig. S9), away from the other crosslinking residues and the WWD domain. CcmD Y17 is highly conserved in γ-proteobacteria (Fig. 7B, S13B) and many, but not all, other classes encode potential heme interacting residues such as Met and His at this position (Fig. 7B; Fig. S13B), suggesting a conserved role for the CcmD periplasmic region in heme interaction.

The driver of heme through the CcmCD heme channel is an open question. CcmABCD is typically compared to ABC transporters, which consist of an ATP-binding cassette (ABC) protein along with integral membrane spanning protein(s) that couple ATP-hydrolysis to solute transport across the membrane (94–98). Yet, CcmABCD does not function as a canonical ABC transporter. Although CcmA binds and hydrolyzes ATP (49, 53, 64), ATP hydrolysis is not required for heme transport. Genetic and biochemical studies demonstrated that in the absence of CcmAB, heme is transported across the membrane and stereospecifically positioned in the CcmC WWD domain (42, 53) and properly attached to CcmE (42, 46, 53, 54) (Fig. S1). Thus, CcmAB, and consequently ATP hydrolysis, are not required for heme transport to the CcmC WWD domain. Rather, studies have shown that ATP hydrolysis via CcmA mediates the release of holoCcmE from the CcmABCD subcomplex (53, 64). A proposed mechanism of holoCcmE release was suggested by Li et al. cryo-EM studies: CcmA ATP hydrolysis mediates conformational changes in CcmB, which in turn results in a switch in CcmC WWD heme liganding from CcmC residues to CcmE Tyr134 and a small ligand such as water. This ligand switch allows for holoCcmE release (49). Further support for holoCcmE release requiring ATP hydrolysis is found from biochemical studies that characterize a stable CcmC/D/holoCcmE complex in the absence of CcmAB (Fig. S1) (45, 46, 54). Therefore, we conclude that CcmCD-mediated heme transport is energy-independent. This is similar to the proposed mechanism of heme transport by System II, CcsBA, which is also proposed to be energy-independent (48).

We speculate that the hydrophobic nature of the heme channel coupled with potential conformational changes in CcmC could accommodate heme transport. Interaction of CcmE with CcmCD or heme attachment to CcmE could mediate the conformational changes and propel heme movement into the channel and across the membrane, similar to the postulated mechanism in CcsBA. Of note, the highly conserved crosslinking residues A97 and I143 bracket the most constrained region of transport, indicating that the conformation of this region is important for the function of CcmC. However, the lack of overall sequence homology in CcmCD encoded in diverse organisms could be explained by hydrophobicity, rather than sequence, being a key component of heme transport, thus suggesting a common mechanism for heme transport in organisms that encode System I.

## MATERIALS AND METHODS

### Bacterial growth conditions

*Escherichia coli* strains were grown in Luria-Bertani (LB, Difco) broth at 37°C, 200 rpm with appropriate antibiotics for selection (carbenicillin, 50 µg/mL; chloramphenicol, 20 µg/mL) and/or inducing reagents (isopropyl-D 1-thiogalactopyranoside [IPTG; GoldBio], 1.0 or 0.1 mM; L-arabinose [Goldbio], 0.2% [wt/vol]). A list of strains and plasmids is provided in Table S2.

### Construction of cysteine variant plasmids

Cloning was performed in *E. coli* NEB-5α. Single amino acid cysteine substitutions and the c-terminal CcmD:flag fusion were engineered via QuikChange II site-directed mutagenesis (Agilent Technologies) and verified via DNA sequencing. Table S3 includes a list of primers and templates.

### Protein purifications

Affinity purifications of GST:CcmCDE(H130A) were performed as previously described (43, 45, 46) with minor modifications. Please see supplemental methods for detailed protocol.

### SDS-PAGE separation and membrane transfer

Protein samples were separated via SDS-PAGE (12.5% or 15%) at 85 V for 15 minutes, then 100 V until the dye front reached the bottom of the apparatus. Heme stain analysis was transferred to 0.2 micron nitrocellulose membrane(s) via wet transfer for 75 minutes at 85 V. Immunoblots were transferred to a 0.45 micron nitrocellulose membrane via semi-dry transfer with BioRad TurboBlot Standard SD manufacturer protocol (up to 1.0 A; 25 V constant) for 30 minutes.

### Heme staining, immunoblotting, quantification, and statistical analysis

Heme staining was performed on 5 or 10 µg of affinity-purified protein samples as previously described using an enhanced chemiluminescent-based development and charge coupled device (CCD) imaging (72, 76). Immunoblots were performed on 5 µg of affinity-purified protein samples and probed with the following antibodies: α-GST (1:20,000) (Invitrogen, PA1-982A), α-CcmE (75) (1:90,000), or α-FLAG (1:30,000) (Sigma, A8592). Protein A peroxidase (Millipore Sigma, P8651) was used as a secondary antibody. Azure Sapphire Biomolecular Imager (Azure, SPC11-0239) was used for imaging. Quantification with AzureSpot Software (Azure, v.2.2.167). The ratio of crosslinked heme was calculated for CcmC/D heme as a fraction of total “free” heme and normalized to wild type. Quantitation was averaged for all biological replicates (CcmD, *n* = 6; CcmC, *n* = 6) and error bars reported as standard deviation. Quantitation of heme retention at the CcmC or CcmD band used the raw heme band signal intensity which was averaged for all biological replicates (CcmC, *n* = 6; CcmD, *n* = 6), error bars report the standard deviation, and an unpaired two-tailed *t*-test was performed with GraphPad Prism v10.4.1.

### UV-vis spectroscopy

UV-vis absorption spectra were collected on a UV-1900i with LabSolutions software (Shimadzu; LabSolutions UV-vis [v1.10]) and performed as described previously (51, 52). Please see supplemental methods for details.

### CcmCD structural predictions and modeling

Prediction of the CcmCD heme channel used the CAVER 3.0.3 PyMOL plugin (71, 99) to model potential heme channels in CcmCD (PDB 7F04). CcmCD hydrophobicity was performed in PyMOL to classify residues as positively charged, negatively charged, hydrophobic, or polar. Prediction of CcmCD structures from other bacteria were obtained in AlphaFold 3 DB version 2024–05-08. PyMOL (version 2.4.1) was used to determine RMSD for predicted structures compared to CcmCD (PDB 7F04). Additional details on structural prediction are provided in the supplemental methods.

### CcmC and CcmD sequence conservation analysis

FASTA amino acid sequences for CcmCD polypeptides were compiled via UniProt (100) and NCBI databases. *E. coli* CcmC (NP_416703.1) amino acid sequence was used as a query for BLASTp (101) searches, and the phylum of interest was selected. All phylum/groups previously reported to encode System I proteins (35) (ɣ-, α-, β-, δ-proteobacteria, plant and protozoal mitochondria, archaea) were included in data collection. The NCBI reference genome designated in UniProt CcmC sequences hits were utilized for all sequence data collection. CcmC and CcmD amino acid sequences within the same operon (within five genes) were identified in the reference genomes to avoid paralogs. Phyla for which both CcmC and CcmD sequences could not be obtained were removed from the analysis. Additional data collection was performed utilizing UniProt “sequence clusters” to identify any additional phyla for which sequence data has revealed the presence of System I CcmC. All sequences were compiled, and reference numbers can be found in Table S3.

Multiple sequence alignments were performed utilizing the UniProt interface (100) of CLUSTAL O (1.2.4) software (102). FASTA sequences were input, and alignment output was compiled. Analysis of multiple sequence alignments (MSA) was performed on the UniProt interface to obtain percent sequence identity. MSA files were uploaded to JalView software (v2.11.4.1) (86) for amino acid conservation and amino acid property conservation analysis.

### *In vivo* cytochrome *c* biogenesis assays and statistical analysis

The CcmC and CcmD cysteine variants were engineered in the full System I pathway (GST:CcmABCD(MBP:E)(F:His)GH), co-expressed with cytochrome *c*_4_:His (pRGK332) in RK103, and cytochrome *c* biogenesis was monitored via heme stain as previously described (75, 76). Please see supplemental methods for details.

### Determination of heme redox potential

Redox potentials were determined as previously described (45), utilizing the modified Massey method (79) developed by Raven et al. (80, 81). Please see supplemental methods for details.

## SUPPLEMENTAL MATERIAL

Supplemental material mbio.03515-24-s0001.pdf Fig. S1-S13; Movie S1; Tables S1-S3; supplemental references.

Movie S1 mbio.03515-24-s0002.mov Model of CcmCD heme transport function.

ASM does not own the copyrights to Supplemental Material that may be linked to, or accessed through, an article. The authors have granted ASM a non-exclusive, world-wide license to publish the Supplemental Material files. Please contact the corresponding author directly for reuse.
