# Supplementary material for: Biochemical mapping reveals a conserved heme transport mechanism via CcmCD in System I bacterial cytochrome c biogenesis
Source: mBio. 2025 Apr 1;16(5):e03515-24. doi: 10.1128/mbio.03515-24 (PMC12077264; doi:10.1128/mbio.03515-24)
Supplement: Supplemental material — Fig. S1-S13; Movie S1; Tables S1-S3; supplemental references. [file mbio.03515-24-s0001.pdf]

Supplemental Information

Title: Biochemical mapping reveals a conserved heme transport mechanism via CcmCD in System I bacterial cytochrome *c* biogenesis

Authors: Alicia N. Kreiman[a], Sarah E. Garner[a], Susan C. Carroll[a], Molly C. Sutherland[a,#]

[a]Department of Biological Sciences, University of Delaware, Newark, Delaware, USA

[#]Corresponding author

Email: msuther@udel.edu

Table of Contents

SUPPLEMENTAL FIGURE 1 3
SUPPLEMENTAL FIGURE 2 4
SUPPLEMENTAL FIGURE 3 5
SUPPLEMENTAL FIGURE 4 6
SUPPLEMENTAL FIGURE 5 8
SUPPLEMENTAL FIGURE 6 10
SUPPLEMENTAL FIGURE 7 11
SUPPLEMENTAL FIGURE 8 12
SUPPLEMENTAL FIGURE 9 13
SUPPLEMENTAL FIGURE 10 14
SUPPLEMENTAL FIGURE 11 16
SUPPLEMENTAL FIGURE 12 17
SUPPLEMENTAL FIGURE 13 18
SUPPLEMENTAL MOVIE 1 20
SUPPLEMENTAL TABLE 1. 21
SUPPLEMENTAL TABLE 2. 23
SUPPLEMENTAL TABLE 3. 25
SUPPLEMENTAL METHODS 27
SUPPLEMENTAL REFERENCES 29

Supplemental Figure 1

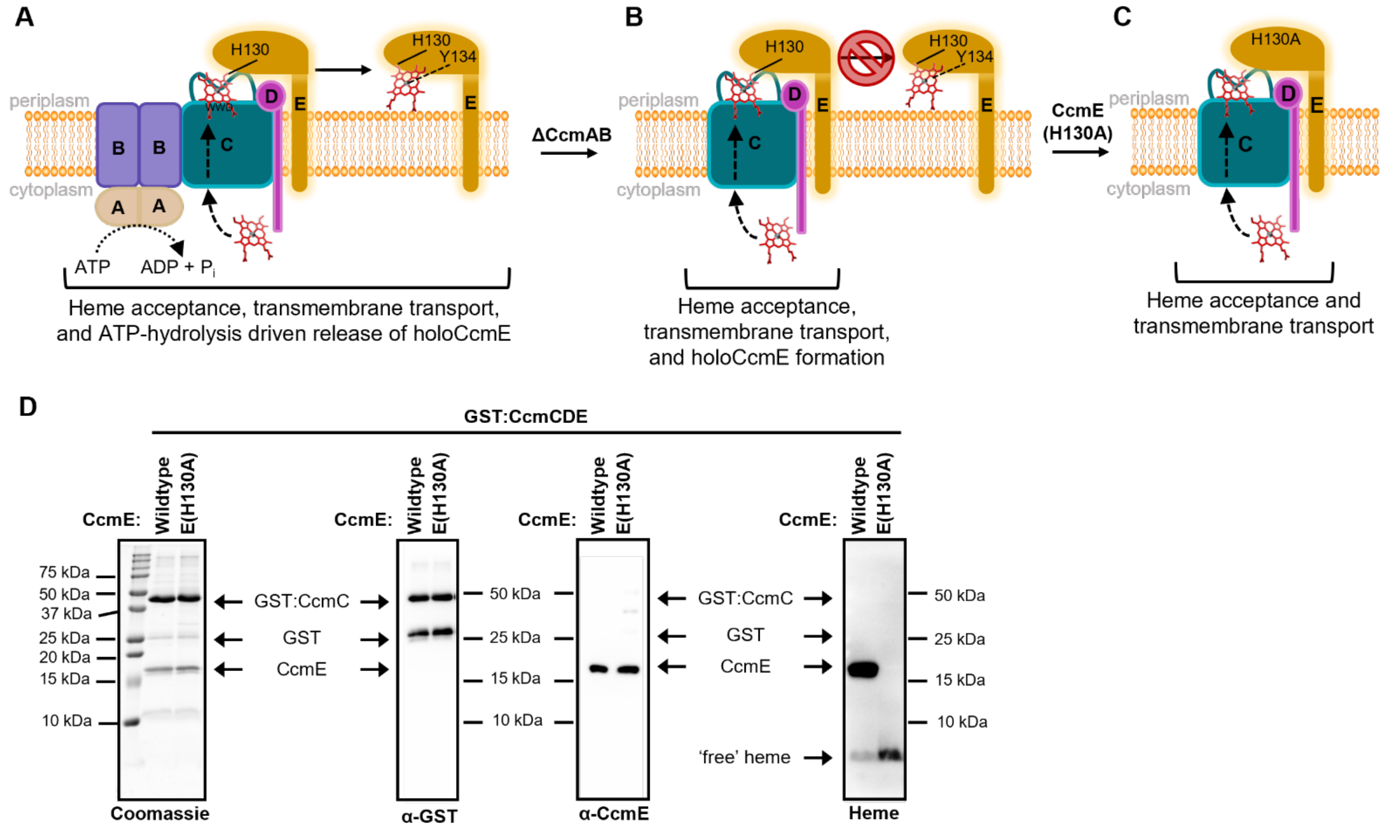

Supplemental Fig. 1. Known functions of CcmABCDE proteins in step 1 of System I. (A) CcmABCDE function as a protein subcomplex for System I. Heme is stereospecifically positioned in the CcmC WWD domain, covalently attached to CcmE at H130 and liganded by Y134. HoloCcmE is released from step 1 via ATP hydrolysis by CcmAB. (B) Deletion of CcmAB prevents release of holoCcmE. However, heme delivery to the CcmC WWD domain is still accomplished and holoCcmE formation occurs with only CcmCDE. (C) HoloCcmE covalent bond formation occurs at H130. Mutation of His130 to Ala inhibits holoCcmE formation, nonetheless heme is still delivered to the CcmC WWD domain. (D) GST:CcmCDE or GST:CcmCDE(H130A) were affinity purified, 5 µg affinity purified protein was separated by SDS-PAGE and assessed for protein stability via Coomassie stain and immunoblotted with antibodies for GST and CcmE. Heme co-purification was assayed via an ECL-based heme stain.

Supplemental Figure 2

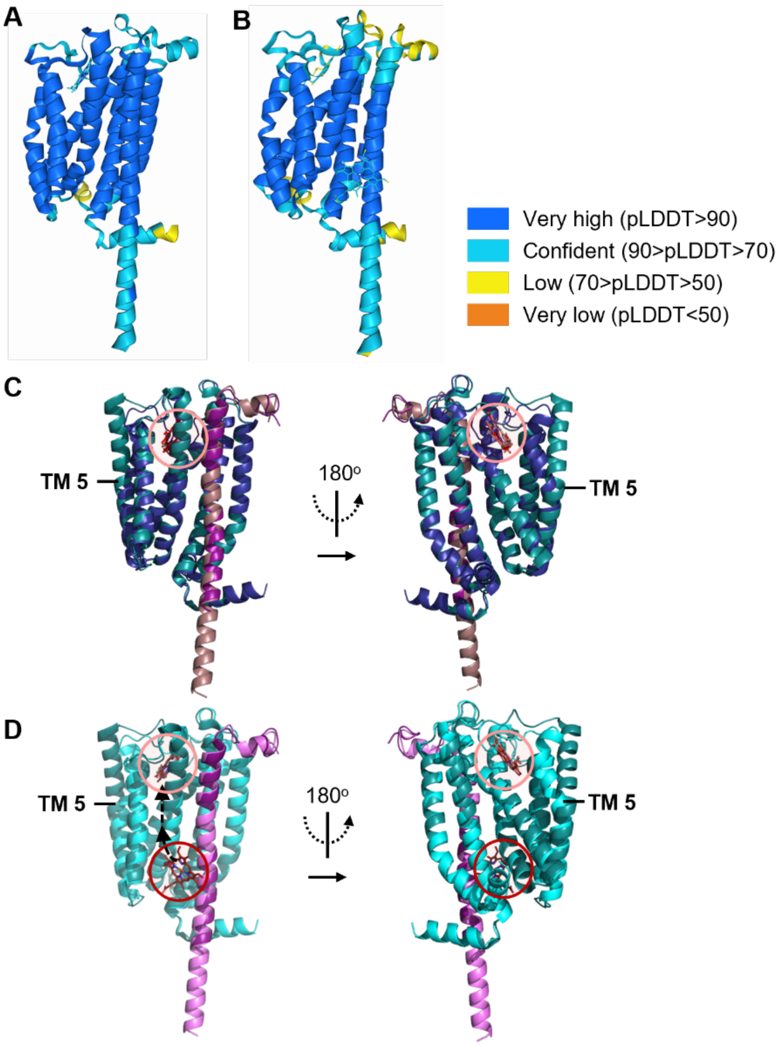

Supplemental Fig. 2. AlphaFold 3 heme placement correlates with Cryo-EM structures (A) AlphaFold 3 (AF3) *E. coli* CcmCD + 1 heme structural prediction confidence values pLDDT. (B) AF3 *E. coli* CcmCD + 2 heme structural prediction confidence values pLDDT. (C) AF3 structure of CcmCD (dark blue/dark pink) + 1 heme molecule (red; salmon circle) superimposed with CcmCD (PDB:7F04) cryo-EM structure (teal/violet); RMSD=0.754. (D) AF3 structure of CcmCD (cyan/light purple) + 2 heme molecules (red) superimposed with CcmCD (PDB:7F04) cryo-EM structure (teal/violet) displaying cytoplasmic heme acceptance domain (red circle), heme transport channel (black arrows), and WWD domain heme (red; salmon circle); RMSD=0.777.

Supplemental Figure 3

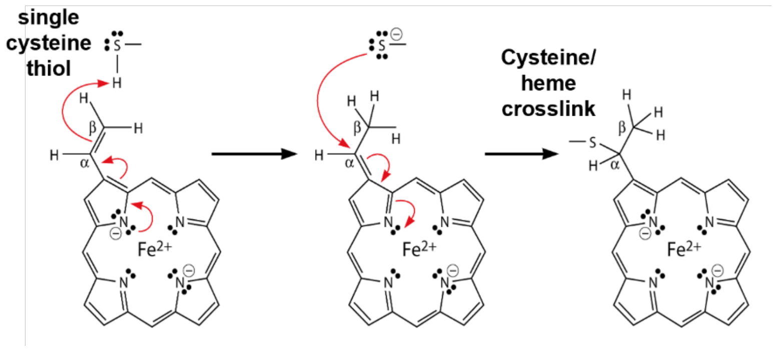

Supplemental Fig. 3. Mechanism of cysteine/heme crosslinking between the cysteine thiol and heme vinyl. A single cysteine thiol is shown for simplicity, red arrows indicate a two electron transfer. The cysteine/heme crosslink is formed via a covalent thioether bond between the alpha carbon of heme and the reduced cysteine thiol. Figure is modified from [1] Sutherland *et al*. 2018. Structurally Mapping Endogenous Heme in the CcmCDE Membrane Complex for Cytochrome c Biogenesis . Journal of Molecular Biology. 430(8):1065-1080.

Supplemental Figure 4

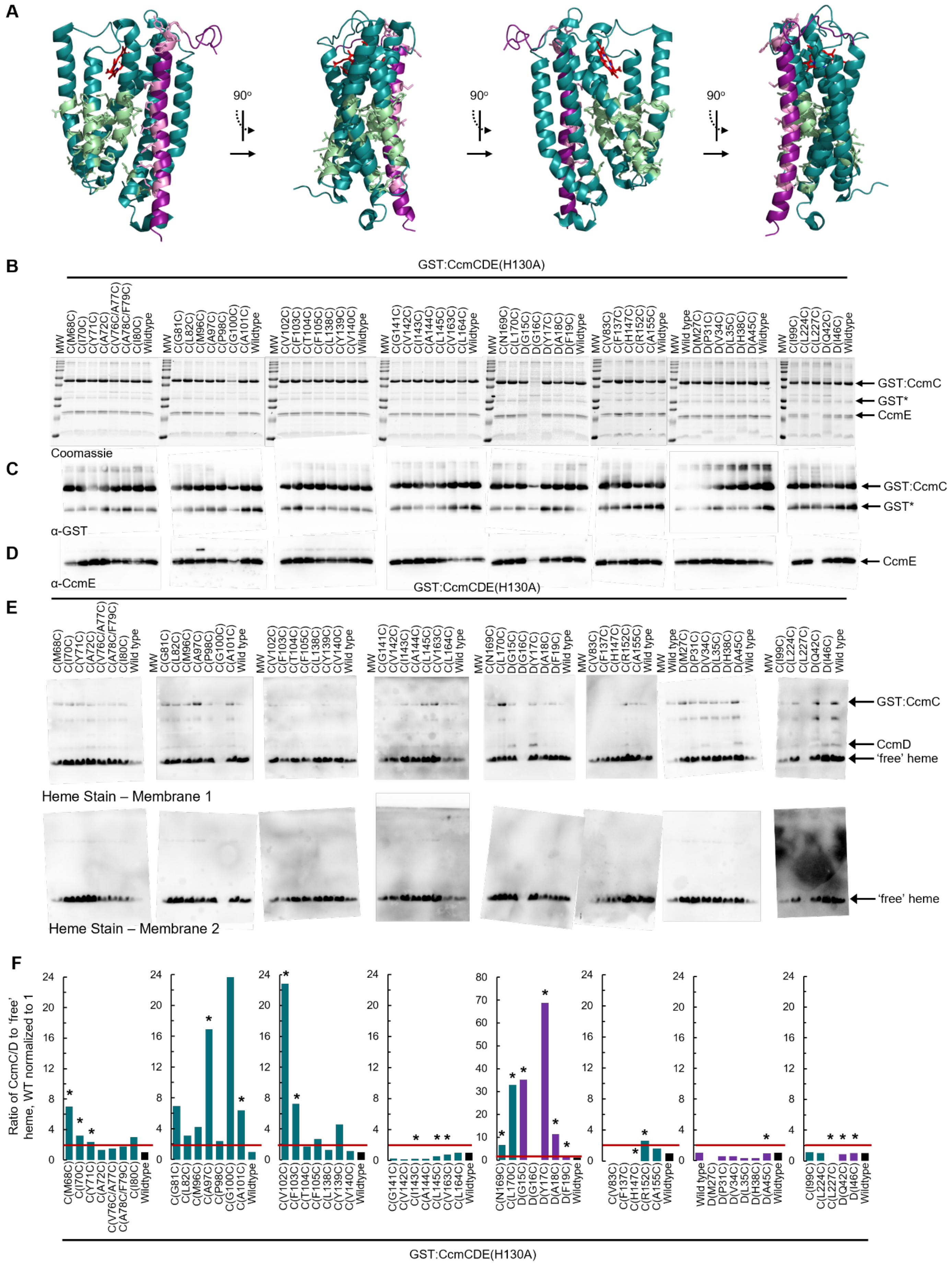

Supplemental Fig. 4. Analysis of fifty-one CcmC and CcmD cysteine variants. (A) CcmCD Cryo-EM structures (PDB:7F04) with cysteine variants indicated in light green (CcmC) or light pink (CcmD) with side chains displayed with sticks. Structure rotated around y-axis at 90° increments. (B-F) Cysteine variants were affinity purified in indicated groups. A wildtype was included in each group as an internal control. Data represents single initial screening of cysteine variants. (B-D) 5 µg of affinity purified protein was separated by SDS-PAGE and assessed for stability of protein complex via (B) coomassie total protein stain, (C) α-GST immunoblot, (D) α-CcmE immunoblot. (E) Heme stain. Free heme can transfer through the nitrocellulose membrane, therefore two membranes are layered to capture the total amount of *b*-type heme that co-purifies with GST:CcmCDE(H130A). (F) Quantitation of heme stain in Fig. S4E; representative of one independent biological replicate. Heme stains were quantified with AzureSpot Software (Azure, v.2.2.167) and the ratio of CcmC/D bound heme to 'free' heme was determined. The wildtype ratio was normalized to 1 and a ratio above 2 (red line) indicated formation of a cysteine/heme crosslink. Variants selected for additional study indicated with an *. Black bars – wild type; teal bars – CcmC; purple bars – CcmD.

Supplemental Figure 5

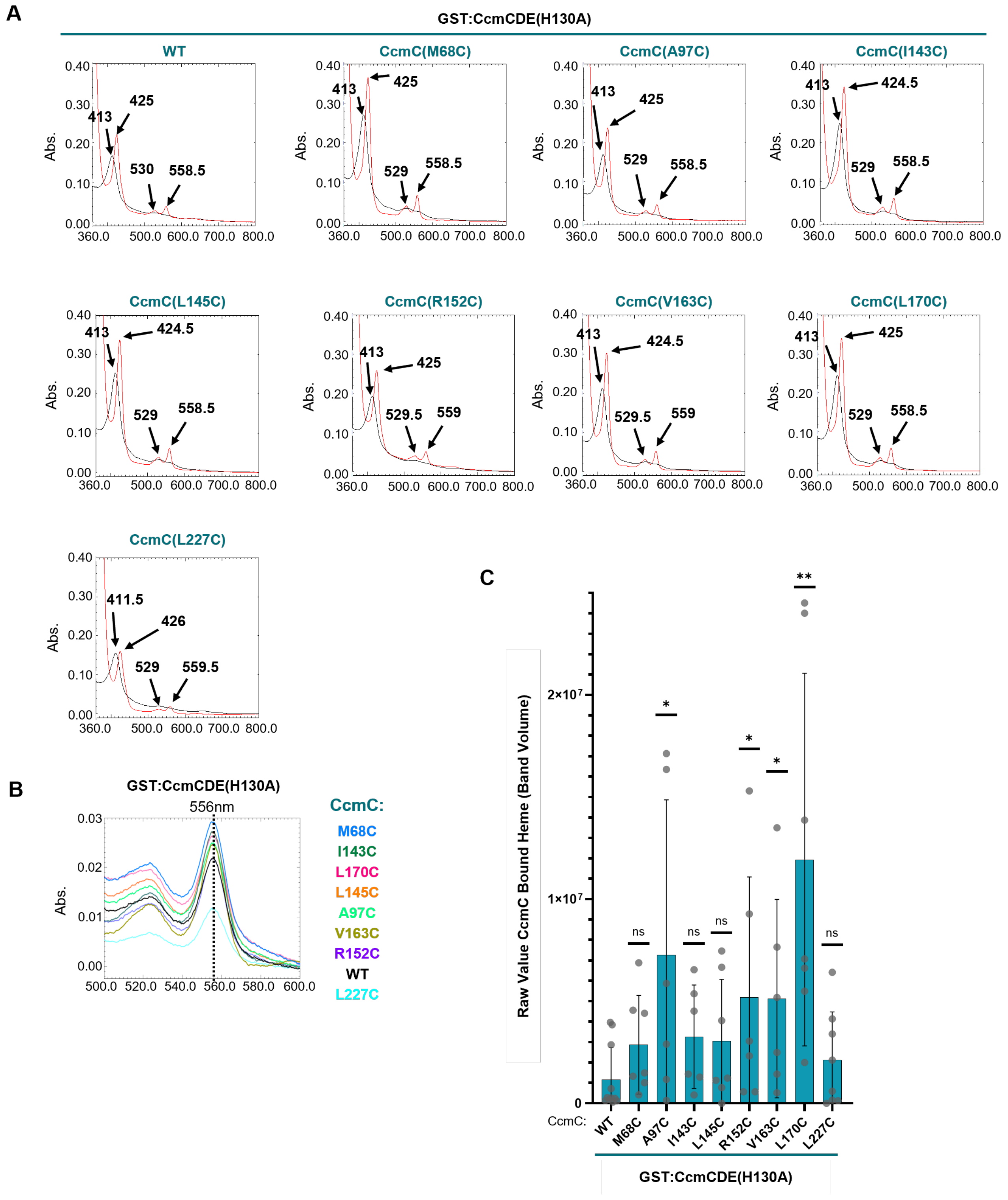

Supplemental Fig. 5. CcmC cysteine variants analysis. (A) UV-vis spectral analysis of as-purified (black) and sodium dithionite reduced (red) purified protein complex, 50 µg affinity purified protein. Wildtype and eight CcmC crosslinking cysteine variants individual UV-vis spectra. Data shown as representative of three independent affinity purifications (B) Pyridine hemochrome reduced spectra, 75 µg affinity purified protein. Wildtype and eight CcmC cysteine crosslinking spectra overlay. Black dotted line - 556 nm wildtype α-peak. Single pyridine hemochrome assay was performed. (C) Formation of the cysteine/heme crosslink has historically used the ratio of cross-linked heme to free heme to account for the variability in cysteine/heme crosslink formation due to the transient nature of heme trafficking coupled with variability in heme co-purification with cytochrome *c* biogenesis proteins (see Fig. 2F). Here we complement this analysis with quantification of only the CcmC heme stained

polypeptide using a two-tailed unpaired t-test. Quantification of the heme stained GST:CcmC polypeptide from six independent purifications (grey dots). Teal bars represent the average GST:CcmC band intensity. Error bars indicate the standard deviation. Statistical analysis was performed with GraphPad Prism (v10.4.1) using a two-tailed unpaired t-test. * $p < 0.05$, ** $p < 0.01$, NS not statistically significant.

Supplemental Figure 6

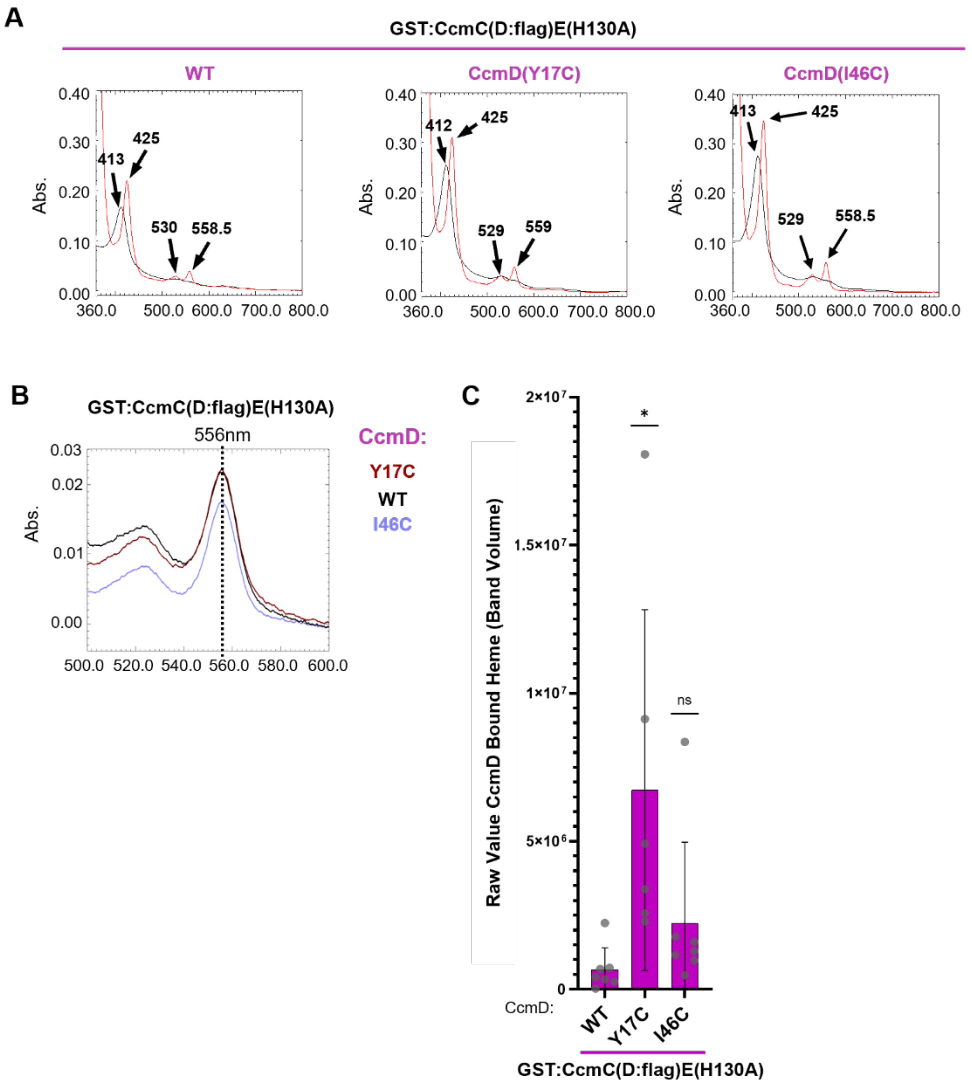

Supplemental Fig. 6. CcmD cysteine variants analysis. (A) UV-vis spectral analysis of as-purified (black) and sodium dithionite reduced (red) purified protein complex, 50 μg affinity purified protein. Wildtype and two CcmD crosslinking cysteine variants individual UV-vis spectra. Data shown as a representative of three independent affinity purifications. (B) Pyridine hemochrome reduced spectra, 75 μg affinity purified protein. Wildtype and two CcmD crosslinking spectra overlay. Black dotted line - 556 nm wildtype α-peak. Single pyridine hemochrome assay was performed. (C) Quantification of the heme stained CcmD:flag polypeptide as described for GST:CcmC in Fig. S5C. Violet bars represent the average CcmD:flag band intensity. Error bars indicate the standard deviation. Grey dots represent six independent purifications. Statistical analysis was performed with GraphPad Prism (v10.4.1) using a two-tailed unpaired t-test. * $p < 0.05$, ** $p < 0.01$, NS not statistically significant.

Supplemental Figure 7

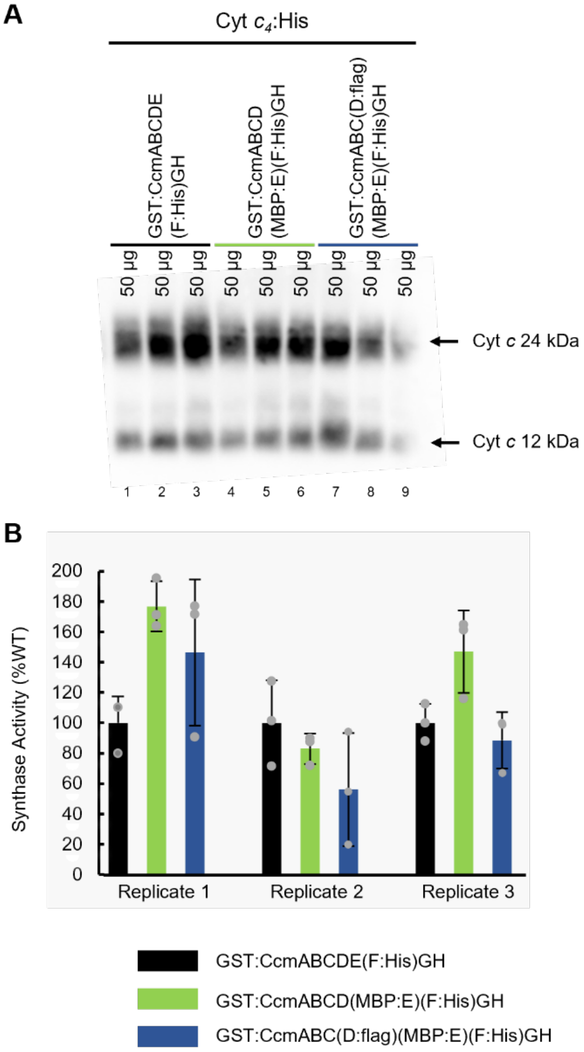

Supplemental Fig. 7. Differentially tagged System I expression constructs are functional for cytochrome *c* biogenesis. The indicated System I construct was co-expressed with cytochrome c_4:His in *E. coli* Δccm. 50 µg cell lysate was separated via SDS-PAGE and levels of cytochrome *c* biogenesis were determined by heme stain. (A) Heme stain, single biological replicate with three technical replicates. (B) Quantitation of heme stain. Three biological replicates, each with three technical replicates (gray dots) are shown. Error bars indicate standard deviation.

Supplemental Figure 8

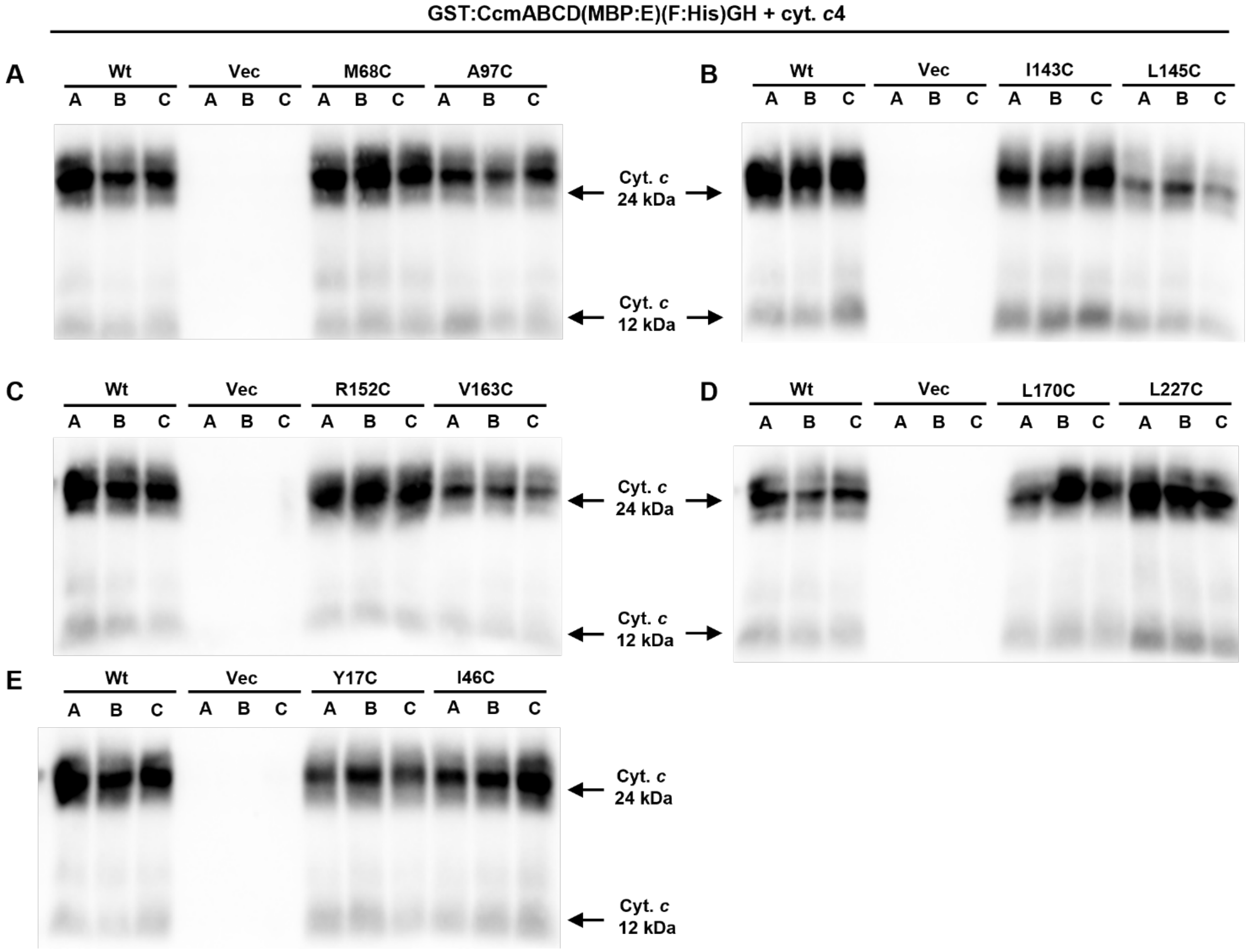

Supplemental Figure 8. Functional analyses of CcmCD cysteine variants. CcmC/D cysteine heme crosslinking variants were co-expressed with cytochrome c_4:His in *E. coli* Δ*ccm.* 50 µg of total cell lysate was separated via SDS-PAGE, transferred to nitrocellulose membrane and heme stained. (A-E) Heme stains of cytochrome *c* biogenesis assay, 50 µg total protein cell lysate. Technical replicates labeled A-C. Data representative of three biological replicates, each containing three technical replicates. The same wildtype and vector samples are loaded on each blot and used as internal controls for quantitation.

Supplemental Figure 9

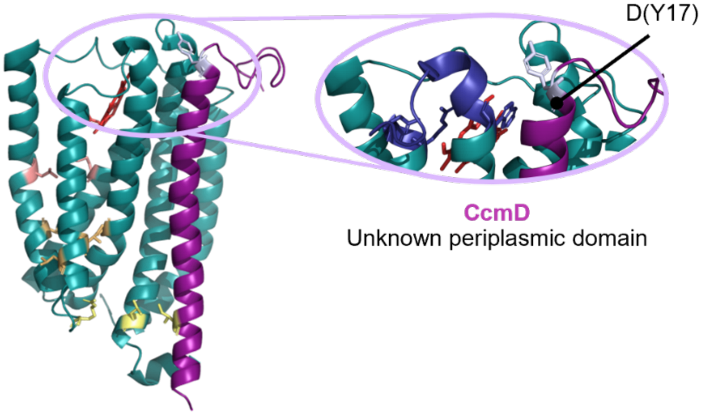

Supplemental Fig. 9. CcmD periplasmic heme interaction domain. Heme handling domain of unknown function CcmD(Y17C) crosslinking residue circled in purple. Image created from PDB:7F04. Cysteine/heme crosslinking variant is labeled.

Supplemental Figure 10

A

Variant	Average (std dev) mV	Ind. Redox mV
CcmD(Y17C)	-131.4 (±9.8)	-137.2, -117.6, -139.5
WT (GST:CDE(H130A))	-120.5 (±0.9)	-119.7, -120.1, -121.8
WT (GST:C(D:flag)E(H130A))	-120.0 (±1.1)	-119.2, -119.3, -121.6
CcmC(L170C)	-115.6 (±6.1)	-121.3, -107.1, -118.4
CcmC(M68C)	-114.1 (±2.7)	-114.2, -117.4, -110.8
CcmC(A97C)	-113.8 (±3.7)	-116.7, -108.6, -116
CcmC(R152C)	-109.7 (±5.5)	-114.7, -102.0, -112.3
CcmD(I46C)	-118.6 (±6.3)	-127.3, -115.7, -112.7

B

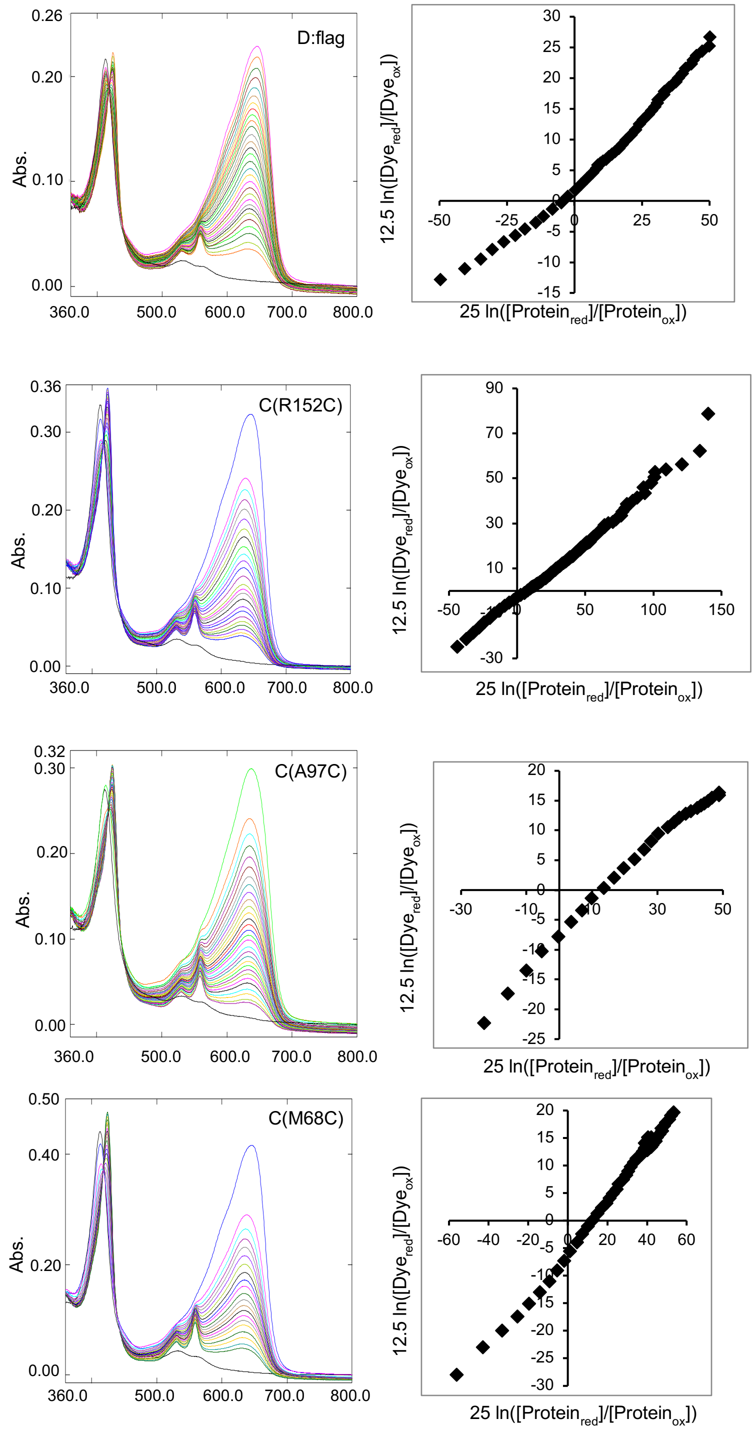

Supplemental Fig. 10. Redox potentials of key CcmCD variants. Heme redox potentials were determined use a modified Massey method. (A) Redox potentials of selected crosslinking variants in each heme-handling/transport domain across CcmCD. Shading indicates domain: purple – periplasmic domain of unknown function, blue – WT WWD domain, red – upper leaflet TMD, orange – lower leaflet TMD, yellow – cytoplasmic heme acceptance domain. (B) UV-vis spectra of redox titration time course and corresponding excel graphs for redox calculations. Representative spectra, excel graphs are shown. All redox titration were performed a minimum of three times, each with independently purified proteins.

Supplemental Figure 11

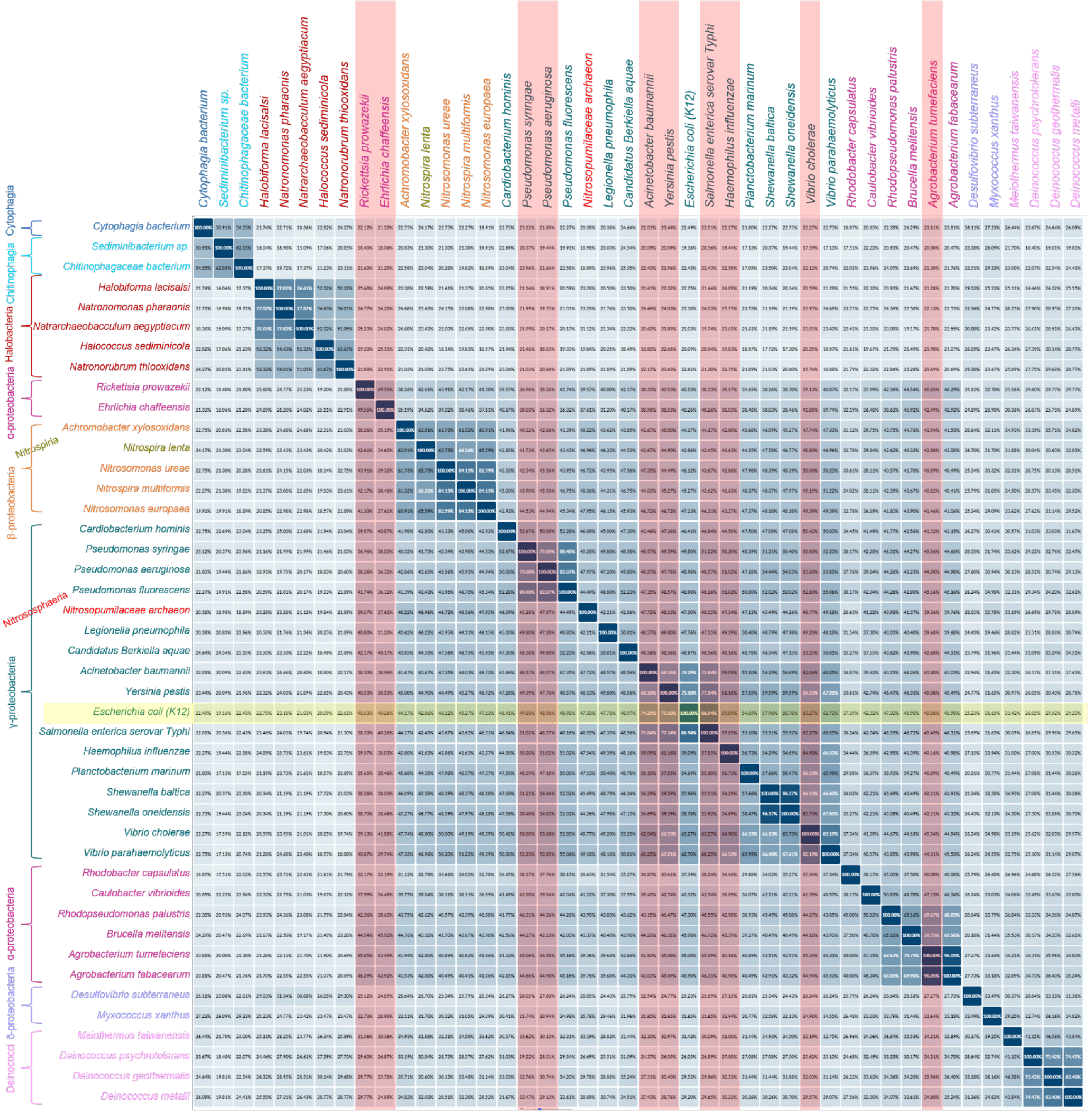

Supplemental Fig. 11. Percent Identity Matrix of CcmC amino acid sequence alignment from *E. coli* + 43 organisms across System I. Sequences aligned utilizing UniProt Clustal O (1.2.4) interface and output determined from alignment. *E. coli* sequence was used as reference sequence for comparison of percent identity (highlighted in yellow). Organisms are labeled by taxonomic class: γ-proteobacteria (teal), β- (orange), α- (maroon), δ- (light purple), Cytophagia (royal blue), Chitinophagia (light blue), Halobacteria (dark red), Nitrospira (gold), Nitrososphaeria (bright red), Deinococci (light pink). Organisms highlighted in red are included in Table 1.

Supplemental Figure 12

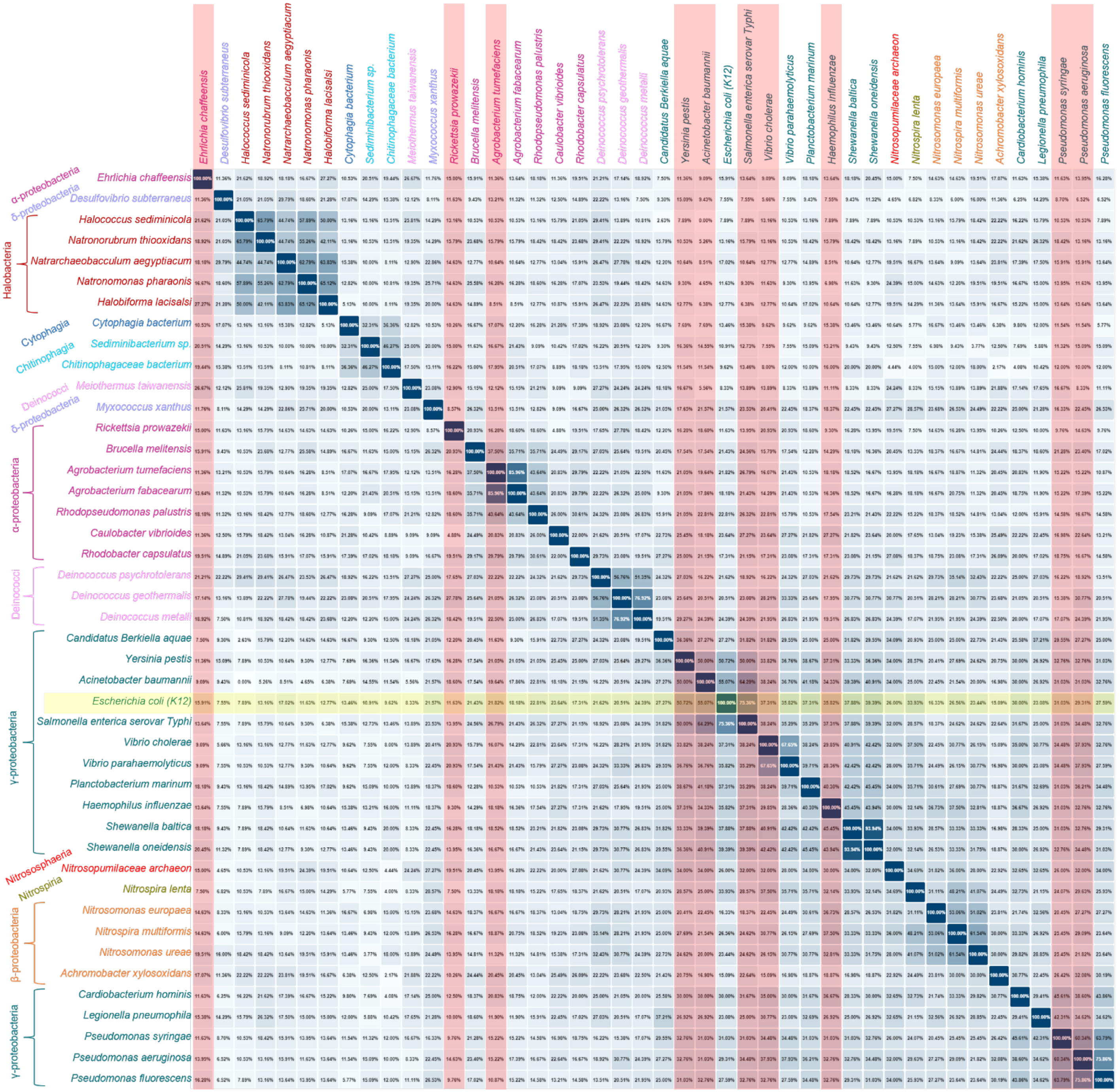

Supplemental Fig. 12. Percent Identity Matrix of CcmD amino acid sequence alignment from *E. coli* + 43 organisms across System I. Sequences aligned utilizing UniProt Clustal O (1.2.4) interface and output determined from alignment. *E. coli* sequence was used as reference sequence for comparison of percent identity (highlighted in yellow). Organisms are labeled by taxonomic class: γ-proteobacteria (teal), β- (orange), α- (maroon), δ- (light purple), Cytophagia (royal blue), Chitinophagia (light blue), Halobacteria (dark red), Nitrospira (gold), Nitrososphaeria (bright red), Deinococci (light pink). Organisms highlighted in red are included in Table 1.

Supplemental Figure 13

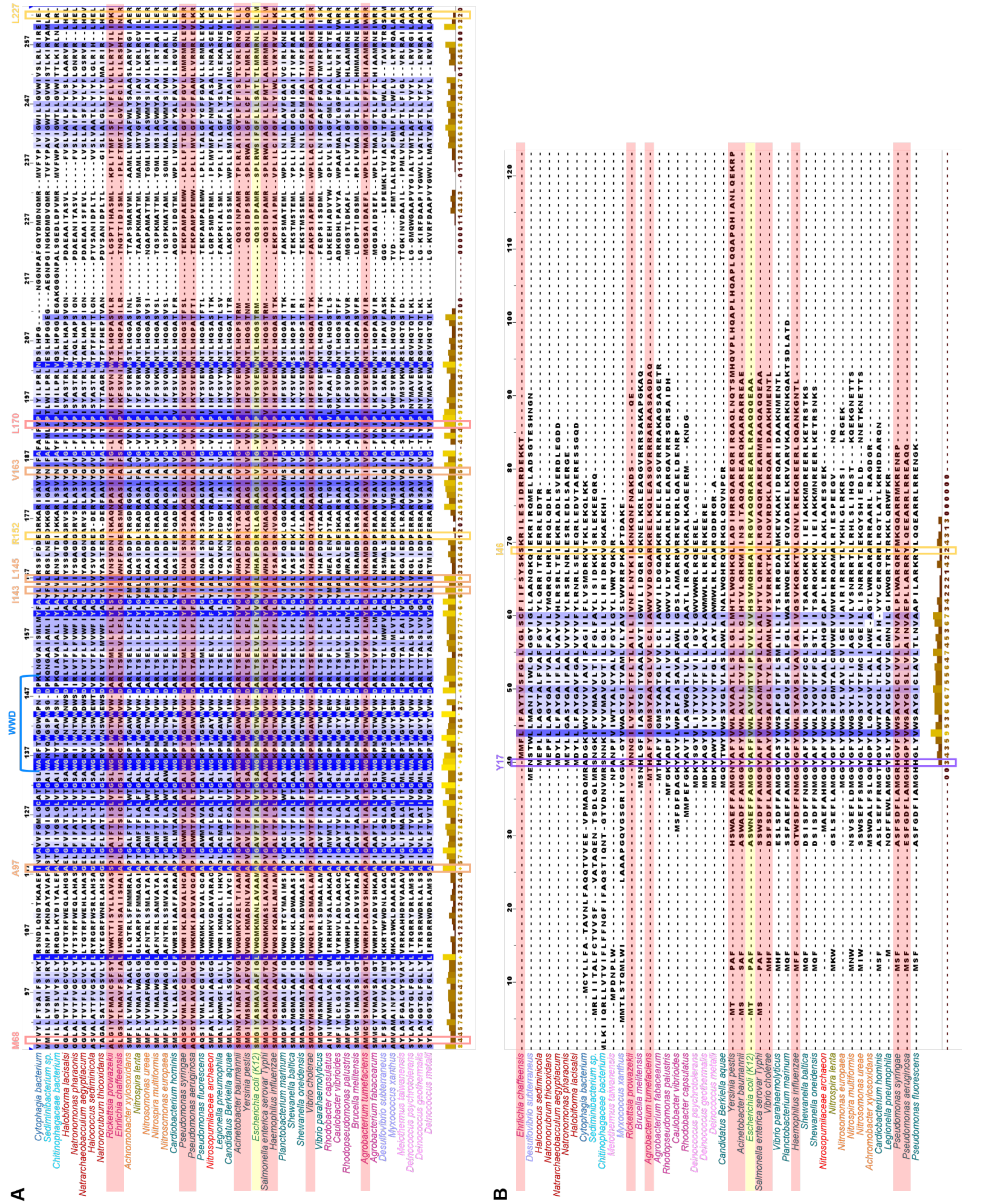

Supplemental Fig. 13. Multiple Sequence Alignment (MSA) of CcmC and CcmD amino acid sequences from *E. coli* and forty-three organisms. Sequences aligned utilizing Clustal O (1.2.4) software through the UniProt interface and output

determined from alignment. *E. coli* CcmC (highlighted in yellow) represents model organism for this study. Conservation per residue calculated in JalView software (v2.11.4.1) and residues are colored by level of conservation >5 (light blue) to fully conserved (royal blue). Amino acids M68 to L227 are displayed with crosslinking residues outlined based on heme transport class defined in Fig. 5 (heme acceptance domain (yellow) C(R152, L227) and D(I46), lower leaflet transmembrane domain (orange) C(A97, I143, L145, V163), upper leaflet transmembrane domain (red) C(M68, L170), periplasmic domain of unknown function (purple) D(Y17). The highly conserved WWD domain is indicated with the blue bracket. Organisms highlighted in red are included in Table 1 (A) CcmC MSA. (B) CcmD MSA.

Supplemental Movie 1

Supplemental Movie 1. Model of CcmCD heme transport. Created from PDB:7F04 in PyMOL (version 2.4.1). Demonstrates predicted transmembrane heme movement through CcmCD based on identified cysteine/heme crosslinking residues (shown as sticks and colored via domain as follows: heme acceptance – yellow, TMD lower leaflet – orange, upper leaflet – red, WWD domain – blue). Briefly, heme is delivered from the cytoplasm to the heme acceptance domain of CcmCD. Next, heme transitions into the core CcmC heme channel composed of TMD 2/3/4/5, travels up the heme transport channel, and finally, is stereospecifically positioned in the WWD domain of CcmC.

Supplemental Table 1.

Supplemental Table 1. Forty-four total organisms containing System I cytochrome *c* biogenesis pathway whose CcmCD sequences were collected for bioinformatic analysis. Pathogens of interest are highlighted in red.

Class	Representative Species	GeneBank ID (reference genome)	CcmC reference #	CcmD reference #	Pathogen REF
gamma-proteobacteria	*Escherichia coli* (K12)	GCA_000005845.1	>NP_416703.1	>NP_416702.1	-
gamma-proteobacteria	*Acinetobacter baumannii*	UFMN01000003.1	>SST03238.1	>SST03237.1	WHO 2024 (Critical)[2] + CDC 2019 (Urgent)[3]
gamma-proteobacteria	*Haemophilus influenzae*	GCF_000931575.1	>WP_044330604.1	WP_005693416.1	WHO 2024 (Medium)[2]
gamma-proteobacteria	*Salmonella enterica* serovar Typhi strain 721597	AAIFQV010000033.1	>ECD7321450.1	>ECD7321449.1	WHO 2024 (High)[2] + CDC 2019 (Serious)[3]
gamma-proteobacteria	*Yersinia pestis* Antiqua	AJJ78172.1	>AJJ80906.1	>AJJ78172.1	NIAID biodefense pathogen list[4]
gamma-proteobacteria	*Pseudomonas syringae* strain MWU 16-30316	SEZV01000001.1	>TFZ38898.1	>TFZ38899.1	Plant pathogen of interest
gamma-proteobacteria	*Candidatus Berkiella aquae*	GCF_001431295.2	>WP_075065853.1	>WP_075065852.1	-
gamma-proteobacteria	*Vibrio cholerae* O1 serovar	CP047301.1	>QJS81051.1	>QJS81052.1	NIAID biodefense pathogen list[4]
gamma-proteobacteria	*Vibrio parahaemolyticus*	GCF_000196095.1	>WP_005457654.1	>WP_005457743.1	-
gamma-proteobacteria	*Shewanella baltica* (strain OS223)	GCF_000178875.2	>WP_006079703.1	>WP_006079702.1	-
gamma-proteobacteria	*Shewanella oneidensis* MR-1	AE014299.2	>AAN53346.1	>AAN53345.1	-
gamma-proteobacteria	*Planctobacterium marinum*	AP027272.1	>BDX07708.1	>BDX07707.1	-
gamma-proteobacteria	*Pseudomonas aeruginosa* PA01	GCF_000006765.1	>NP_250168.1	>NP_250169.1	WHO 2024 (High)[2] + CDC 2019 (Serious)[3]
gamma-proteobacteria	*Pseudomonas fluorescens*	CP148752.1	>WXR69450.1	>WXR69451.1	-
gamma-proteobacteria	*Legionella pneumophila*	GCA_001753085.1	>WP_011213329.1	>WP_011945969.1	-
gamma-proteobacteria	*Cardiobacterium hominis*	GCF_900637305.1	>WP_004140696.1	>WP_004140695.1	-
beta-proteobacteria	*Nitrospira multiformis* ATCC 25196	GCF_000196355.1	>WP_011380556.1	>WP_011380555.1	-
beta-proteobacteria	*Nitrosomonas europaea* ATCC 19718	GCF_000009145.1	>WP_011111378.1	>WP_234988995.1	-
beta-proteobacteria	*Nitrosomonas ureae*	WP_062559647.1	>WP_062559647.1	>SNX58885.1	-
beta-proteobacteria	*Achromobacter xylosoxidans*	GCF_016728825.1	>WP_006384724.1	>WP_020927183.1	-
alpha-proteobacteria	*Rickettsia prowazekii* str. Chernikova	GCF_000277165.1	>WP_004596831.1	>AFE49388.1	NIAID biodefense pathogen list[4]
alpha-proteobacteria	*Ehrlichia chaffeensis*	CP007480.1 GCA_000632965.1	>WP_006010829.1	>AHX10971.1	NIAID biodefense pathogen list[4]
alpha-proteobacteria	*Brucella melitensis* bv. 1 str. 16M	GCA_000007125.1	>AAL53032.1	>AAL53031.1	-
alpha-proteobacteria	*Agrobacterium tumefaciens* strC58	NC_003062.2	>WP_006310864.1	>WP_006310865.1	Plant pathogen of interest
alpha-proteobacteria	*Agrobacterium fabacearum* P4	GCF_000442985.1	>WP_025594583.1	>WP_013636916.1	-
alpha-proteobacteria	*Rhodopseudomonas palustris*	GCF_016584445.1	>WP_011155773.1	>WP_012493979.1	-
alpha-proteobacteria	*Caulobacter vibrioides (Caulobacter crescentus CB15)*	GCF_000022005.1	>YP_002519159.1	>YP_002519160.1	-
alpha-proteobacteria	*Rhodobacter capsulatus*	GCF_000021865.1	>WP_013067511.1	>WP_013067512.1	-
delta-proteobacteria	*Myxococcus xanthus*	GCF_000012685.1	>WP_011553301.1	>WP_011553300.1	-
delta-proteobacteria	*Desulfovibrio subterraneus*	GCF_027558435.1	>WP_174406143.1	>WP_174405168.1	-
Nitrospiria	*Nitrospira lenta*	GCF_900403705.1	>WP_121987557.1	>WP_121987556.1	-
Chitinophagia	*Sediminibacterium sp*	PGCO01000013.1	>PJE45959.1	>PJE45958.1	-
Chitinophagia	*Chitinophagaceae bacterium*	JACDBU010000083.1	>MBA2249319.1	>MBA2249320.1	-
Cytophagia	*Cytophagia bacterium*	REAB01000229.1	>TAG34495.1	>TAG34505.1	-

Deinococci	*Deinococcus geothermalis*	GCF_000196275.1	>WP_011530378.1	>WP_083807144.1	-
Deinococci	*Deinococcus metalli*	JACHFK010000001.1	>MBB5374879.1	>GHF33041.1	-
Deinococci	*Deinococcus psychrotolerans*	CP034183.1	>AZI43147.1	>AZI43146.1	-
Deinococci	*Meiothermus taiwanensis*	QWKX01000004.1	>RIH79654.1	>RIH79653.1	-
Nitrososphaeria	*Nitrosopumilaceae archaeon*	UYNY00000000.3	>CAI2287500.1	>CAI2287499.1	-
Halobacteria	*Natronomonas pharaonis* (strain ATCC 35678)	CR936257.1	>WP_011322744.1	>WP_011322743.1	-
Halobacteria	*Natrarchaeobaculum aegyptiacum*	CP019893.1	>ARS90949.1	>ARS90948.1	-
Halobacteria	*Halobiforma lacisalsi*	NZ_CP019285.1	>WP_007141830.1	>WP_007141831.1	-
Halobacteria	*Halococcus sediminicola*	GCF_000755245.1	>WP_237561118.1	>WP_079977575.1	-
Halobacteria	*Natronorubrum thiooxidans*	NZ_FTNR01000001.1	>WP_076607571.1	>WP_076607570.1	-

Supplemental Table 2.

Supplemental Table 2. Relevant strains and plasmids utilized in this study.

Plasmid, strain	Description	Reference
E. coli		
RK103	*E. coli* MG1655 *Δccm::kanR* , deleted for all *ccm* genes	Feissner *et al*, 2006[5]
NEB 5-α	fhuA2 Δ(argF-lacZ)U169 phoA glnV44 Φ80 Δ(lacZ)M15 gyrA96 recA1 relA1 endA1 thi-1 hsdR17	
Plasmid		
pRGK375	pGEX WT GST:CcmCDE	Richard-Fogal *et al*, 2009[6]
pRGK380	pGEX WT GST:CcmCDE(H130A)	Richard-Fogal *et al*, 2009[6]
pMCS1243	pGEX GST:CcmC(D:flag)E(H130A)	This study
pRGK332	pBAD cytochrome *c4*:His	Feissner *et al*, 2006[5]
	**CcmC cysteine variants**	
pMCS612	pGEX GST:CcmC(M68C) DE(H130A)	This study
pMCS613	pGEX GST:CcmC(I70C) DE(H130A)	This study
pMCS614	pGEX GST:CcmC(Y71C) DE(H130A)	This study
pMCS615	pGEX GST:CcmC(A72C) DE(H130A)	This study
pMCS427	pGEX GST:CcmC(V76C/A77C)DE(H130A)	This study
pMCS425	pGEX GST:CcmC(A78C/F79C)DE(H130A)	This study
pMCS420	pGEX GST:CcmC(I80C)DE(H130A)	This study
pMCS422	pGEX GST:CcmC(G81C)DE(H130A)	This study
pMCS407	pGEX GST:CcmC(L82C)DE(H130A)	This study
pMCS424	pGEX GST:CcmC(V83C)DE(H130A)	This study
pMCS426	pGEX GST:CcmC(M96C)DE(H130A)	This study
pMCS408	pGEX GST:CcmC(A97C)DE(H130A)	This study
pMCS440	pGEX GST:CcmC(P98C) DE(H130A)	This study
pMCS409	pGEX GST:CcmC(I99C)DE(H130A)	This study
pMCS410	pGEX GST:CcmC(G100C)DE(H130A)	This study
pMCS616	pGEX GST:CcmC(A101C) DE(H130A)	This study
pMCS617	pGEX GST:CcmC(V102C) DE(H130A)	This study
pMCS618	pGEX GST:CcmC(F103C) DE(H130A)	This study
pMCS619	pGEX GST:CcmC(T104C) DE(H130A)	This study
pMCS620	pGEX GST:CcmC(F105C) DE(H130A)	This study
pMCS621	pGEX GST:CcmC(F137C) DE(H130A)	This study
pMCS411	pGEX GST:CcmC(L138C)DE(H130A)	This study
pMCS392	pGEX GST:CcmC(Y139C)DE(H130A)	This study
pMCS443	pGEX GST:CcmC(V140C) DE(H130A)	This study
pMCS431	pGEX GST:CcmC(G141C)DE(H130A)	This study
pMCS413	pGEX GST:CcmC(V142C)DE(H130A)	This study
pMCS415	pGEX GST:CcmC(I143C)DE(H130A)	This study
pMCS419	pGEX GST:CcmC(A144C)DE(H130A)	This study
pMCS417	pGEX GST:CcmC(L145C)DE(H130A)	This study
pMCS1119	pGEX GST:CcmC(H147C)DE(H130A)	This study
pMCS1108	pGEX GST:CcmC(R152C)DE(H130A)	This study
pMCS1109	pGEX GST:CcmC(A155C)DE(H130A)	This study
pMCS421	pGEX GST:CcmC(V163C)DE(H130A)	This study
pMCS423	pGEX GST:CcmC(L164C)DE(H130A)	This study
pMCS622	pGEX GST:CcmC(N169C)DE(H130A)	This study
pMCS623	pGEX GST:CcmC(L170C) DE(H130A)	This study
pMCS1110	pGEX GST:CcmC(L224C)DE(H130A)	This study
pMCS1111	pGEX GST:CcmC(L227C)DE(H130A)	This study
	**CcmD cysteine variants**	
pMCS412	pGEX GST:CcmCD(G15C)E(H130A)	This study
pMCS414	pGEX GST:CcmCD(G16C)E(H130A)	This study
pMCS393	pGEX GST:CcmCD(Y17C)E(H130A)	This study
pMCS418	pGEX GST:CcmCD(A18C)E(H130A)	This study
pMCS416	pGEX GST:CcmCD(F19C)E(H130A)	This study
pMCS1112	pGEX GST:CcmCD(M27C)E(H130A)	This study
pMCS1113	pGEX GST:CcmCD(P31C)E(H130A)	This study
pMCS1114	pGEX GST:CcmCD(V34C)E(H130A)	This study
pMCS1115	pGEX GST:CcmCD(L35C)E(H130A)	This study
pMCS1120	pGEX GST:CcmCD(H38C)E(H130A)	This study
pMCS1116	pGEX GST:CcmCD(Q42C)E(H130A)	This study
pMCS1117	pGEX GST:CcmCD(A45C)E(H130A)	This study
pMCS1118	pGEX GST:CcmCD(I46C)E(H130A)	This study
pMCS1256	pGEX GST:CcmC(D(Y17C):flag)E(H130A)	This study
pMCS1257	pGEX GST:CcmC(D(I46C):flag)E(H130A)	This study
	**CcmC/D cysteine variants – full System I**	
pRGK386	pGEX GST:CcmABCDE(F:His)GH	Richard-Fogal *et al*, 2009[6]
pMCS250	pGEX GST:CcmABCD(MBP:E)(F:His)GH	This study

pMCS1311	pGEX GST:CcmABC(D:flag)(MBP:E)(F:His)GH	This Study
pMCS1247	pGEX GST:CcmABC(M68C)D(MBP:E)F:His(GH)	This study
pMCS1248	pGEX GST:CcmABC(A97C)D(MBP:E)F:His(GH)	This study
pMCS1249	pGEX GST:CcmABC(I143C)D(MBP:E)F:His(GH)	This study
pMCS1250	pGEX GST:CcmABC(L145C)D(MBP:E)F:His(GH)	This study
pMCS1251	pGEX GST:CcmABC(R152C)D(MBP:E)F:His(GH)	This study
pMCS1252	pGEX GST:CcmABC(V163C)D(MBP:E)F:His(GH)	This study
pMCS1253	pGEX GST:CcmABC(L170C)D(MBP:E)F:His(GH)	This study
pMCS1226	pGEX GST:CcmABC(L227C)D(MBP:E)F:His(GH)	This study
pMCS1235	pGEX GST:CcmABCD(Y17C)(MBP:E)F:His(GH)	This study
pMCS1233	pGEX GST:CcmABCD(I46C)(MBP:E)F:His(GH)	This study

Supplemental Table 3.

Supplemental Table 3. Relevant primers and templates utilized in this study.

Oligonucleotide	Sequence (5' - 3')	*Purpose*	*Template*
MSP773	gcggcgatctggtcgtgcggcatttatgcatcaatgg	pMCS612, pMCS1247 cloning	pRGK380, pMCS250
MSP774	ccattgatgcataaatgccgcacgaccagatcgccgc	pMCS612, pMCS1247 cloning	pRGK380, pMCS250
MSP775	gcgatctggtcgatgggcatttgtgcatcaatggcag	pMCS614 cloning	pRGK380
MSP776	ctgccattgatgcacaaatgcccatcgaccagatcgc	pMCS614 cloning	pRGK380
MSP0215	gcatttatgcatcaatggcatgttgcgcgtttattggccttg	pMCS427 cloning	pRGK380
MSP0216	caaggccaataaacgcgcaacatgccattgatgcataaatgc	pMCS427 cloning	pRGK380
MSP0213	ttatgcatcaatggcagtggcatgctgtattggccttgtctggcagatg	pMCS425 cloning	pRGK380
MSP0214	catctgccagacaaggccaatacagcatgccactgccattgatgcataa	pMCS425 cloning	pRGK380
MSP0185	catcaatggcagtggcagcgttttgtggccttgtctg	pMCS420 cloning	pRGK380
MSP0186	cagacaaggccacaaaacgctgccactgccattgatg	pMCS420 cloning	pRGK380
MSP0187	gcagtggcagcgtttatttgccttgtctggc	pMCS422 cloning	pRGK380
MSP0188	gccagacaaggcaaataaacgctgccactgc	pMCS422 cloning	pRGK380
MSP0169	agtggcagcgtttattggctgtgtctggcagatgaaaatg	pMCS407 cloning	pRGK380
MSP0170	cattttcatctgccagacacagccaataaacgctgccact	pMCS407 cloning	pRGK380
MSP0191	ggcggtggcggcgtgcgcccccattggtg	pMCS426 cloning	pRGK380
MSP0192	gcaccaatgggggcgcacgccgccaccgccagg	pMCS426 cloning	pRGK380
MSP0171	ggtggcggcgatgtgccccattggtgcc	pMCS408, pMCS1248 cloning	pRGK380, pMCS250
MSP0172	ggcaccaatggggcacatcgccgccacc	pMCS408, pMCS1248 cloning	pRGK380, pMCS250
MSP0193	ggcggcgatggcctgcattggtgccgtg	pMCS440 cloning	pRGK380
MSP0194	cacggcaccaatgcaggccatcgccgcc	pMCS440 cloning	pRGK380
MSP0287	taaacacggcacaaatgggggccatcgcc	pMCS410 cloning	pRGK380
MSP0288	ggcgatggcccccatttgtgccgtgttta	pMCS410 cloning	pRGK380
MSP0197	gaactggtgctgctgttttgctatgtgggtgtgattgcc	pMCS411 cloning	pRGK380
MSP0198	ggcaatcacacccacatagcaaaacagcagcaccagttc	pMCS411 cloning	pRGK380
MSP0217	atcacacccacacacaaaaacagcagcaccagttca	pMCS392 cloning	pRGK380
MSP0218	tgaactggtgctgctgtttttgtgtgtgggtgtgat	pMCS392 cloning	pRGK380
MSP0199	cttctgaactggtgctgctgtttttgtattgcggtgtgattgccctg	pMCS443 cloning	pRGK380
MSP0200	cagggcaatcacaccgcaatacaaaaacagcagcaccagttcagaag	pMCS443 cloning	pRGK380
MSP0175	tgctgtttttgtatgtgtgtgtgattgccctgtgg	pMCS431 cloning	pRGK380
MSP0176	ccacagggcaatcacacacacatacaaaaacagca	pMCS431 cloning	pRGK380
MSP0201	gctgtttttgtatgtgggttgcattgccctgtggcacgcct	pMCS413 cloning	pRGK380
MSP0202	aggcgtgccacagggcaatgcaacccacatacaaaaacagc	pMCS413 cloning	pRGK380
MSP0203	tttgtatgtgggtgtgtgtgccctgtggcacgcc	pMCS415, pMCS1249 cloning	pRGK380, pMCS250
MSP0204	ggcgtgccacagggcacacacacccacatacaaa	pMCS415, pMCS1249 cloning	pRGK380, pMCS250
MSP0205	gctgtttttgtatgtgggtgtgatttgcctgtggcacgcc	pMCS419 cloning	pRGK380
MSP0206	ggcgtgccacaggcaaatcacacccacatacaaaaacagc	pMCS419 cloning	pRGK380
MSP0207	tgtgggtgtgattgcctgctggcacgccttcgacg	pMCS417, pMCS1250 cloning	pRGK380, pMCS250
MSP0208	cgtcgaaggcgtgccagcaggcaatcacacccaca	pMCS417, pMCS1250 cloning	pRGK380, pMCS250
MSP0209	gtgcggcaggtatcctgtgcctgattggcgtggtgaa	pMCS421 cloning	pRGK380
MSP0210	ttcaccacgccaatcaggcacaggatacctgccgcac	pMCS421 cloning	pRGK380
MSP0211	gcggcaggtatcctggtgtgcattggcgtggtgaatctg	pMCS423 cloning	pRGK380
MSP0212	cagattcaccacgccaatgcacaccaggatacctgccgc	pMCS423 cloning	pRGK380
MSP769	cctggtgctgattggcgtggtgaattgcccgattattcattactc	pMCS623, pMCS1253 cloning	pRGK380, pMCS250
MSP770	gagtaatgaataatcgggcaattcaccacgccaatcagcaccagg	pMCS623, pMCS1253 cloning	pRGK380, pMCS250
MSP763	gcaatgggcggttgtgcctttttgtctggctggcgg	pMCS393, pMCS1256, pMCS1235 cloning	pRGK380, pMCS1243, pMCS250
MSP764	ccgccagccagacaaaaaggcacaaccgcccattgc	pMCS393, pMCS1256, pMCS1235 cloning	pRGK380, pMCS1243, pMCS250
MSP795	cctggaatgaatttttcgcaatgtgcggttacgcctttttgtctggc	pMCS412 cloning	pRGK380
MSP796	gccagacaaaaaggcgtaaccgcacattgcgaaaaattcattccagg	pMCS412 cloning	pRGK380
MSP771	tgaatttttcgcaatgggctgttacgcctttttgtctggc	pMCS414 cloning	pRGK380
MSP772	gccagacaaaaaggcgtaacagcccattgcgaaaaattca	pMCS414 cloning	pRGK380
MSP0183	gggcggttacgcctgttttgtctggctgg	pMCS416 cloning	pRGK380
MSP0184	ccagccagacaaaacaggcgtaaccgccc	pMCS416 cloning	pRGK380
MSP765	gcaatgggcggttactgctttttgtctggctggcgg	pMCS418 cloning	pRGK380
MSP766	ccgccagccagacaaaaagcagtaaccgcccattgc	pMCS418 cloning	pRGK380
MSP0704	ggtcgtcgaaggcgcaccacagggcaatcacaccc	pMCS1119 cloning	pRGK380
MSP0705	gggtgtgattgccctgtggtgcgccttcgacgacc	pMCS1119 cloning	pRGK380
MSP0718	ccgccagacggcagtcgtcgaaggc	pMCS1108, pMCS1251 cloning	pRGK380, pMCS250
MSP0719	gccttcgacgactgccgtctggcgg	pMCS1108, pMCS1251 cloning	pRGK380, pMCS250
MSP0720	ctgccgcacggccgcacagacggcggtcg	pMCS1109 cloning	pRGK380
MSP0721	cgaccgccgtctgtgcggccgtgcggcag	pMCS1109 cloning	pRGK380
MSP755	ccatcagcaaaatgcaattacgcatccgcatc	pMCS1110 cloning	pRGK380
MSP756	gatgcggatgcgtaattgcattttgctgatgg	pMCS1110 cloning	pRGK380
MSP0724	cggcgttttccatgcacaaaatcaaattacgc	pMCS1111, pMCS1226 cloning	pRGK380, pMCS250
MSP0725	gcgtaatttgattttgtgcatggaaaaacgccg	pMCS1111, pMCS1226 cloning	pRGK380, pMCS250

MSP0726	ccagcggaataacggtgcacaccaccgccagccag	pMCS1112 cloning	pRGK380
MSP0727	ctggctggcggtggtgtgcaccgttattccgctgg	pMCS1112 cloning	pRGK380
MSP0728	ccaaaaccaccaggcaaataacggtcatcaccaccgcc	pMCS1113 cloning	pRGK380
MSP0729	ggcggtggtgatgaccgttatttgcctggtggttttgg	pMCS1113 cloning	pRGK380
MSP0730	ccgagtgcacgaccaaacacaccagcggaataacgg	pMCS1114 cloning	pRGK380
MSP0731	ccgttattccgctggtgtgtttggtcgtgcactcgg	pMCS1114 cloning	pRGK380
MSP0732	ccgagtgcacgacgcaaaccaccagcggaataac	pMCS1115 cloning	pRGK380
MSP0733	gttattccgctggtggtttgcgtcgtgcactcgg	pMCS1115 cloning	pRGK380
MSP0710	gcgatgttgcatcaccgagcacacgaccaaaaccaccag	pMCS1120 cloning	pRGK380
MSP0711	ctggtggttttggtcgtgtgctcggtgatgcaacatcgc'	pMCS1120 cloning	pRGK380
MSP754	cagaattgcgcgatggcacatcaccgagtgcacgac	pMCS1116 cloning	pRGK380
MSP735	gtcgtgcactcggtgatgtgccatcgcgcaattctgcg	pMCS1116 cloning	pRGK380
MSP736	gccacgccacgcagaatgcagcgatgttgcatcaccg	pCMS1117 cloning	pRGK380
MSP737	cggtgatgcaacatcgctgcattctgcgtggcgtggc	pMCS 1117 cloning	pRGK380
MSP738	ccacgccacgcagacatgcgcgatgttgcatc	pMCS1118, pMCS1257. pMCS1233 cloning	pRGK380, pMCS1243, pMCS250
MSP739	gatgcaacatcgcgcatgtctgcgtggcgtgg	pMCS1118, pMCS1257, pMCS1233 cloning	pRGK380, pMCS1243, pMCS250
MSP712	gtggcagcgtttattggcctttgctggcagatg	pMCS424 cloning	pRGK380
MSP713	catctgccagcaaaggccaataaacgctgccac	pMCS424 cloning	pRGK380
MSP714	cgatggccccctgtggtgccgtgtttacc	pMCS409 cloning	pRGK380
MSP715	ggtaaacacggcaccacagggggccatcg	pMCS409 cloning	pRGK380
MSP716	ctggtgctgctgtgtttgtatgtgggtgtg	pMCS621 cloning	pRGK380
MSP717	cacacccacatacaaacacagcagcaccag	pMCS621 cloning	pRGK380
MSP999	actgccattgatgcataacagcccatcgaccagatcgc	pMCS613 cloning	pRGK380
MSP1000	gcgatctggtcgatgggctgttatgcatcaatggcagt	pMCS613 cloning	pRGK380
MSP1001	cgctgccactgccattgagcaataaatgcccatcgaccagatc	pMCS615 cloning	pRGK380
MSP1002	gatctggtcgatgggcatttattgctcaatggcagtggcagcg	pMCS615 cloning	pRGK380
MSP1003	aataaaggtaaacacgcaaccaatgggggccatcgcc	pMCS616 cloning	pRGK380
MSP1004	ggcgatggcccccattggttgcgtgtttacctttatt	pMCS616 cloning	pRGK380
MSP1005	ccagggcaataaaggtaaagcaggcaccaatgggggccatc	pMCS617 cloning	pRGK380
MSP1006	gatggcccccattggtgcctgctttacctttattgccctgg	pMCS617 cloning	pRGK380
MSP1007	cccattggtgccgtgtgtacctttattgccctg	pMCS618 cloning	pRGK380
MSP1008	cagggcaataaaggtacacacggcaccaatggg	pMCS618 cloning	pRGK380
MSP1009	gtaaccagggcaataaagcaaaacacggcaaccaatggg	pCMS619 cloning	pRGK380
MSP1010	cccattggtgccgtgttttgctttattgccctggttac	pMCS619 cloning	pRGK380
MSP1011	ggtaaccagggcaatacaggtaaacacggcacc	pMCS620 cloning	pRGK380
MSP1012	ggtgccgtgtttacctgtattgccctggttacc	pMCS620 cloning	pRGK380
MSP1013	gtaatgaataatcggcagacacaccacgccaatcagcacc	pMCS622 cloning	pRGK380
MSP1014	ggtgctgattggcgtggtgtgtctgccgattattcattac	pMCS622 cloning	pRGK380
MSP777	cgtgcggcaggtatcctgtgcctgattggcgtggtgaa	pMCS1252 cloning	pMCS250
MSP778	ttcaccacgccaatcaggcacaggatacctgccgcacg	pMCS1252 cloning	pMCS250
MSP767	cgtgctgcgcaacagcaggaggctgcagactacaaggatgacgacgat aagtgaatgaatattcgccgtaaaaaccgcttgtgg	pMCS1243 cloning	pRGK380
MSP768	ccacaagcggtttttacggcgaatattcattcacttatcgtcgtcatccttgta gtctgcagcctcctgctgttgcgcagcacg	pMCS1243 cloning	pRGK380

Supplemental Methods

Protein purifications
Affinity purifications of GST:CcmCDE(H130A) were performed as previously described[6–8] with minor modifications. RK103 *E. coli* Δ*ccm* was used for recombinant protein expression. Briefly, 10 mL starter culture was back diluted 1:100 in 1 L LB with appropriate antibiotics and grown at 37°C and 200 rpm for four hours. Cultures were induced with 1 mM IPTG, grown for an additional ~16-18 hours, harvested by centrifugation and cell pellets were stored at -80°C. Cells were resuspended in GST buffer (4.3 mM Na_2HPO_4, 1.5 mM KH_2PO_4, 2.7 mM KCl, 140 mM NaCl, pH7.3), supplemented with 1 mM phenylmethansulfonyl fluoride (PMSF, Sigma-Aldrich, P-470-10) and 1 mg/mL egg white lysozyme (Sigma-Aldrich, L-040-10). Cells were lysed by sonication (BransonSFX250 sonicator), cleared of cell debris by centrifugation at 10,000 rpm for 1 hour at 4°C, followed by separation of soluble and membrane fractions via high-speed ultracentrifugation at 100,000*g* for 45 minutes at 4°C. Membrane pellets were stored at -80°C, then solubilized in GST buffer with 1% n-dodecyl-β-d-maltopyranoside (DDM, GoldBio, DDM25) and affinity purified with glutathione agarose (Pierce, G-250-5) via batch purification for 2 hours at 4°C. Columns were washed by gravity flow, eluted in 4 mL GST buffer supplemented with 0.02% DDM and 10 mM L-glutathione (Sigma-Aldrich) and then concentrated in a 30 kDa filter. Note: no exogenous heme was added to cultures or during purification. Protein concentrations determined by Bradford Assay (Sigma-Aldrich, PI23200).

UV-vis spectroscopy
UV-vis absorption spectra were collected on a UV-1900i with LabSolutions software (Shimadzu; LabSolutions UV-vis [v1.10]) and performed as described previously[9,10]. Briefly, 50 µg affinity purified protein was assayed in the purification buffer. Reduction was performed with excess sodium hydrosulfite powder (Sigma, 157-953). Quantitation of relative heme co-purification levels were determined via as-purified Soret comparison. UV-vis spectra are representative of three independent protein purifications. Pyridine hemochrome spectra were performed as previously described [11]. Briefly, 75 ug purified protein, resulting in a Soret absorbance of at least 0.2 was used. The total volume was adjusted to 120 µl with GST buffer supplemented with 0.02% DDM. NaOH and pyridine were added to a final concentration of 100 mM NaOH and 20% pyridine (v/v). Samples were reduced as described above. Spectra were recorded from 500-600 nm. Pyridine hemochrome assays were performed once.

***In vivo* cytochrome *c* biogenesis assays and statistical analysis**
The CcmC and CcmD cysteine variants were engineered in the full System I pathway (GST:CcmABCD(MBP:E)(His:F)GH), co-expressed with cytochrome c_4:His (pRGK332) in RK103 and cytochrome *c* biogenesis was monitored via heme stain as previously described[12,13]. Briefly, 1 mL of saturated overnight cultures were back diluted 1:6 into 5 mL LB with appropriate antibiotics and grown for 3 hours at 37°C and 200 rpm. Protein expression was induced with 0.1 mM IPTG and 0.2% arabinose and grown for 3 hours at 37°C and 200 rpm. Cell pellets were collected by centrifugation at 3700 rpm for 10 minutes and frozen at -80°C. Bacterial Protein Extraction Reagent (B-PER, Thermo Scientific, PI78248) was used to lyse cells per manufacturer's instructions. 50 µg protein from total cell lysate was separated by SDS-PAGE and cytochrome *c* biogenesis (i.e., heme attachment to cytochrome *c*) was analyzed via enhanced chemiluminescent heme stain[14]. Heme stains were quantified with AzureSpot Software (Azure, v.2.2.167) and wildtype was normalized to 100%. Unpaired two-tailed t-test were performed with GraphPad Prism v10.4.1. A minimum of three biological replicates, each containing three technical replicates, were performed.

CcmCD structural predictions and modeling
Prediction of the CcmCD heme channel: CAVER 3.0.3 PyMOL plugin[15,16] was utilized to model potential heme channels in CcmCD (PDB:7F04). The predicted cytoplasmic heme domain (Fig. 1B) was selected as a starting point; standard input values were utilized except the probe radius was decreased to 0.5 and the maximum distance (A) was increased to 5.

Analysis of CcmCD hydrophobicity: PyMOL (version 2.4.1) was used to classify and color residues in CcmCD as positively charged (blue: Arg, Lys, His), negatively charged (red: Asp, Glu), hydrophobic (grey: Ala, Gly, Val, Ile, Leu, Phe, Met, Pro) or polar (pink: Asn, Gln, Thr, Ser, Cys, Tyr, Trp). Analysis displayed as a cartoon with an overlay of transparent surface.

Predicted structures of CcmCD from other bacteria: Predicted structures of *E. coli* (K12) CcmCD with 1 or 2 heme ligands (CcmC:NP_416703.1 CcmD:NP_416702.1), *P. syringae* (CcmC: TFZ38898.1 CcmD: TFZ38899.1), *P. aeruginosa* (CcmC: NP_250168.1 CcmD: NP_250169.1), *R. prowazekii* (CcmC: WP_004596831.1 CcmD: AFE49388.1), and *A. tumefaciens* (CcmC: WP_006310864.1 CcmD: WP_006310865.1) were obtained in AlphaFold 3 DB version 2024-05-08. Most predicted structures had a high level of confidence (per residue confidence score, pLDDT, greater than 90) (Fig. S2). Predicted structural CIF files and PDB:7F04 of *E. coli* CcmABCD[17] were uploaded and compared using PyMOL (version 2.4.1). Alignment of predicted structures to target CcmCD (PDB:7F04) were performed in PyMOL, one to one with outliers removed over 5 cycles and RMSD calculated.

Determination of heme redox potential

Redox potentials were determined as previously described[8], utilizing the modified Massey method[18] developed by Raven and colleagues[19,20]. Please see Supplemental Methods for details. Briefly, the cysteine variants in the GST:CcmCDE(H130A) background were affinity purified, and heme redox potentials were determined via simultaneous reduction of the purified protein complex with a dye of known reduction potential in bulk solution. Nile blue was used as the reference dye and acted as a mediator [20]. Nile blue is suitable for use with heme complexes of the System I pathway and does not interfere with UV-vis spectral reading at ~420 nm, the Soret peak of heme [8]. Likewise, heme does not interfere with the reference wavelength of 630 nm for the reduction of Nile Blue. Samples were placed in a Coy anaerobic chamber and allowed to equilibrate with N_2 (95%) and H_2 (5%) for approximately 3 hours. All assays were performed under anaerobic conditions in GST buffer supplemented with 0.02% DDM, 625 μM xanthine (Millipore Sigma, X4002), and Nile blue chloride (E_m = -116mV) [21] as the reference dye. The reaction was initiated via the addition of 100 – 125 μM xanthine oxidase (Millipore Sigma X4376). Scanning UV-vis spectra was collected from 360 – 800 nm every minute utilizing a UV-1900i Shimadzu spectrophotometer with LabSolutions software (UV-vis [v1.10]). Reduction of heme was monitored via the absorbance change at 420 nm and 423 nm, and reduction of Nile blue was monitored via the absorbance change at 630 nm. Data analysis was completed in Excel utilizing the Nerst equation: one-electron reduction of heme with E = 25 mV*ln(b $heme_{red}$/b $heme_{ox}$) and two-electron reduction of Nile blue with E = 12.5 mV*ln(dye_{red}/dye_{ox}). Given the assumption that at the time point when the reduced/oxidized ratio is (A_{ox}- A_t)/(A_t-A_{red}) with A_{ox} being the absorbance from the most oxidized spectrum and A_{red} being the absorbance of the most reduced spectrum; the ratios of reduced/oxidized heme and dye were calculated for each absorbance spectral data point collected and represent the molar concentrations of ratio of reduced/oxidized state of heme and dye. If the ratio was greater than 10 or less than 0.1 as compared to the next recorded data point, then those points were excluded from the analysis. E_{heme} vs E_{dye} was plotted, and the y-intercept value represents the difference between the redox potential of Nile blue (-116 mV) and the redox potential of the purified protein. This difference was used to calculate the redox potential of the purified protein. Potentials are given versus the standard hydrogen electrode. Reduction potentials were calculated as the average of three independent assays with the standard deviation reported.

Supplemental References

1. Sutherland, M. C., Jarodsky, J. M., Ovchinnikov, S., Baker, D. & Kranz, R. G. Structurally Mapping Endogenous Heme in the CcmCDE Membrane Complex for Cytochrome c Biogenesis. *J. Mol. Biol.* **430**, 1065–1080 (2018).
2. WHO bacterial priority pathogens list, 2024: Bacterial pathogens of public health importance to guide research, development and strategies to prevent and control antimicrobial resistance. https://www.who.int/publications/i/item/9789240093461.
3. CDC. 2019 Antibiotic Resistance Threats Report. *Antimicrobial Resistance* https://www.cdc.gov/antimicrobial-resistance/data-research/threats/index.html (2024).
4. NIAID Biodefense Pathogens | NIAID: National Institute of Allergy and Infectious Diseases. https://www.niaid.nih.gov/research/niaid-biodefense-pathogens (2024).
5. Feissner, R. E., Richard-Fogal, C. L., Frawley, E. R. & Kranz, R. G. ABC transporter-mediated release of a haem chaperone allows cytochrome c biogenesis. *Molecular Microbiology* **61**, 219–231 (2006).
6. Richard-Fogal, C. L. *et al.* A conserved haem redox and trafficking pathway for cofactor attachment. *The EMBO Journal* **28**, 2349–2359 (2009).
7. Sutherland, M. C., Jarodsky, J. M., Ovchinnikov, S., Baker, D. & Kranz, R. G. Structurally Mapping Endogenous Heme in the CcmCDE Membrane Complex for Cytochrome c Biogenesis. *J Mol Bio* **430**, 1065–1080 (2018).
8. Sutherland, M. C., Rankin, J. A. & Kranz, R. G. Heme Trafficking and Modifications during System I Cytochrome *c* Biogenesis: Insights from Heme Redox Potentials of Ccm Proteins. *Biochemistry* **55**, 3150–3156 (2016).
9. Grunow, A. L., Carroll, S. C., Kreiman, A. N. & Sutherland, M. C. Structure-function analysis of the heme-binding WWD domain in the bacterial holocytochrome c synthase, CcmFH. *mBio* e0150923 (2023) doi:10.1128/mbio.01509-23.
10. Yeasmin, T., Carroll, S. C., Hawtof, D. J. & Sutherland, M. C. Helicobacter pylori and Campylobacter jejuni bacterial holocytochrome c synthase structure-function analysis reveals conservation of heme binding. *Commun Biol* **7**, 984 (2024).

11. Berry, E. A. & Trumpower, B. L. Simultaneous determination of hemes a, b, and c from pyridine hemochrome spectra. *Anal. Biochem.* **161**, 1–15 (1987).

12. Feissner, R. E. *et al.* Recombinant cytochromes c biogenesis systems I and II and analysis of haem delivery pathways in Escherichia coli. *Mol. Microbiol.* **60**, 563–577 (2006).

13. Kreiman, A. N., Yeasmin, T. & Sutherland, M. C. Recombinant Biogenesis and Analysis of Cytochrome c Species. in *Iron Metabolism* (ed. Khalimonchuk, O.) vol. 2839 195–211 (Springer US, New York, NY, 2024).

14. Feissner, R., Xiang, Y. & Kranz, R. G. Chemiluminescent-based methods to detect subpicomole levels of c-type cytochromes. *Anal. Biochem.* **315**, 90–94 (2003).

15. Chovancova, E. *et al.* CAVER 3.0: A Tool for the Analysis of Transport Pathways in Dynamic Protein Structures. *PLoS Comput Biol* **8**, e1002708 (2012).

16. Pavelka, A. *et al.* CAVER: Algorithms for Analyzing Dynamics of Tunnels in Macromolecules. *IEEE/ACM Trans Comput Biol Bioinform* **13**, 505–517 (2016).

17. Li, J. *et al.* Structures of the CcmABCD heme release complex at multiple states. *Nat Commun* **13**, 6422 (2022).

18. Massey, V. in *Flavins and Flavoproteins* 59–66 (Walter de Gruyter & Co., New York, 1991).

19. Efimov, I. *et al.* The redox properties of ascorbate peroxidase. *Biochemistry* **46**, 8017–8023 (2007).

20. Efimov, I. *et al.* A simple method for the determination of reduction potentials in heme proteins. *FEBS Lett.* **588**, 701–704 (2014).

21. Clark, W. M. *Oxidation-Reduction Potentials of Organic Systems*. (The Williams & Wilkins Company, Baltimore, 1960).
